# Novel *N*-(Heterocyclylphenyl)benzensulfonamide Sharing an Unreported Binding Site with T-Cell Factor 4 at the β-Catenin Armadillo Repeats Domain as an Anticancer Agent

**DOI:** 10.1021/acsptsci.3c00092

**Published:** 2023-07-03

**Authors:** Marianna Nalli, Laura Di Magno, Yichao Wen, Xin Liu, Michele D’Ambrosio, Michela Puxeddu, Anastasia Parisi, Jessica Sebastiani, Andrea Sorato, Antonio Coluccia, Silvia Ripa, Fiorella Di Pastena, Davide Capelli, Roberta Montanari, Domiziana Masci, Andrea Urbani, Chiara Naro, Claudio Sette, Viviana Orlando, Sara D’Angelo, Stefano Biagioni, Chiara Bigogno, Giulio Dondio, Arianna Pastore, Mariano Stornaiuolo, Gianluca Canettieri, Te Liu, Romano Silvestri, Giuseppe La Regina

**Affiliations:** † Laboratory affiliated to Istituto Pasteur Italia—Fondazione Cenci Bolognetti, Department of Drug Chemistry and Technologies, Sapienza University of Rome, Piazzale Aldo Moro 5, I-00185 Rome, Italy; ‡ Laboratory affiliated to Istituto Pasteur Italia—Fondazione Cenci Bolognetti, Department of Molecular Medicine Sapienza, University of Rome, Viale Regina Elena 291, I-00161 Rome, Italy; § Shanghai Geriatric Institute of Chinese Medicine, Shanghai University of Traditional Chinese Medicine, 365 South Xiangyang Road, 200031 Shanghai, China; ∥ Department of Dermatology, Yueyang Hospital of Integrated Traditional Chinese and Western Medicine, Shanghai University of Traditional Chinese Medicine, 200437 Shanghai, China; ⊥ CNR—Institute of Crystallography, Via Salaria—km 29.300, Monterotondo, 00015 Rome, Italy; # Department of Basic Biotechnological Sciences, Intensivological and Perioperative Clinics, Catholic University of the Sacred Heart, Largo Francesco Vito 1, 00168 Rome, Italy; ∇ Department of Biology and Biotechnologies “Charles Darwin”, Piazzale Aldo Moro 5, I-00185 Roma, Italy; ○ Aphad SrL, Via della Resistenza 65, 20090 Buccinasco, Italy; ◆ Department of Pharmacy, University of Naples “Federico II”, Via Domenico Montesano, 49, 80131 Naples, Italy; ¶ GSTeP-Organoids Research Core Facility, Fondazione Policlinico Universitario A. Gemelli, IRCCS, 00168 Rome, Italy

**Keywords:** β-catenin, c-MYC, T-cell factor, colorectal cancer, sulfonamide, crystal structure

## Abstract

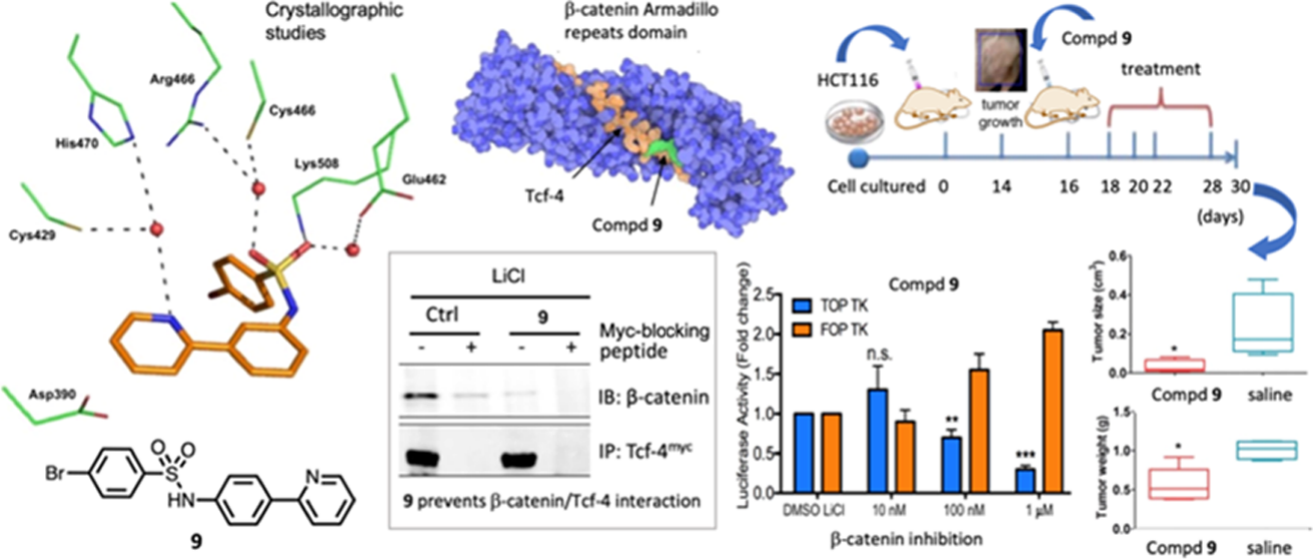

Despite intensive efforts, no inhibitors of the Wnt/β-catenin signaling pathway have been approved so far for the clinical treatment of cancer. We synthesized novel *N*-(heterocyclylphenyl)benzenesulfonamides as β-catenin inhibitors. Compounds **5**–**10** showed strong inhibition of the luciferase activity. Compounds **5** and **6** inhibited the MDA-MB-231, HCC1806, and HCC1937 TNBC cells. Compound **9** induced in vitro cell death in SW480 and HCT116 cells and in vivo tumorigenicity of a human colorectal cancer line HCT116. In a co-immunoprecipitation study in HCT116 cells transfected with Myc-tagged T-cell factor 4 (Tcf-4), compound **9** abrogated the association between β-catenin and Tcf-4. The crystallographic analysis of the β-catenin Armadillo repeats domain revealed that compound **9** and Tcf-4 share a common binding site within the hotspot binding region close to Lys508. To our knowledge, compound **9** is the first small molecule ligand of this region to be reported. These results highlight the potential of this novel class of β-catenin inhibitors as anticancer agents.

Wingless/integrase-1 (Wnt)/β-catenin signaling, a pathway that regulates tissue homeostasis, evades its tight control in many human diseases. In physiological conditions and in the absence of Wnt ligands, the pathway is kept inactive by means of the β-catenin destruction complex. As part of this complex, β-transducing repeats-containing proteins (β-TrCP) ubiquitinate β-catenin and promote its proteasomal degradation. Mutations in gene coding for members of the destruction complex, mainly adenomatous polyposis coli (APC), or in the β-catenin gene itself all cause inhibition of β-catenin ubiquitin-proteasome degradation and lead to the accumulation of β-catenin. Escaping from its degradation destiny, β-catenin translocates into the nucleus and recruits transcriptional co-activators, causing chromatin modification and prompting the expression of the T-cell factor (TCF) and lymphoid enhancer-binding factor (LEF) target genes.^1−5^

The Wnt/β-catenin signaling pathway appears to be dysregulated in tumor initiation, proliferation, and metastasis. The induction of cell death by interfering with the Wnt/β-catenin signaling pathway presents an attractive opportunity to design new chemotherapeutic agents. Activation of Wnt/β-catenin involves the binding to TCF and additional co-transcription factors. Hence, direct interfering with these binding interactions shows promising potential in inhibiting the aberrant activation of the Wnt/β-catenin signaling pathway in cancer.^6^

In cancer cells, β-catenin promotes transcription of the oncogenes, *Axin-2*, *c-Myc*, and *Cyclin-D1*, ultimately promoting the growth of several types of cancers, including colon cancer, hepatocellular carcinoma, pancreatic cancer, lung cancer, and ovarian cancer.^7^ In the last decades, intensive efforts to discover specific inhibitors of the Wnt/β-catenin signaling pathway have been documented. These inhibitors include small molecules, peptides, antibodies, and RNAi molecules that have been shown to target intracellular or extracellular Wnt/β-catenin pathway members.^8^ Small molecules can be categorized as those (i) inhibiting β-catenin/Tcf interactions, (ii) antagonizing transcriptional co-activators, (iii) binding to the PDZ domain of Disheveled (DVL), or (iv) inhibiting other Wnt-interconnected pathways.^8^ However, to our knowledge none of these inhibitors have been approved so far for the clinical treatment of cancer.

Chemotherapy and radiotherapy are the principal treatments for patients with early-stage non-metastatic colorectal cancer (CRC) or CRC at stages I to III (cancer growth through the mucosa—inner lining), whereas systemic chemotherapy is restricted to patients with metastatic CRC (mCRC).^9^ The 5-year survival rate is 90% for patients with early CRC, 70% for patients with locally advanced CRC, and 15% for patients with mCRC. There are no general treatments that can provide a successful outcome in every treated CRC patient; moreover, acquired drug resistance may add difficulty. Novel therapeutic approaches are being researched thanks to the most recent pathological and immunological advances. New agents may improve survival and give hope to CRC patients suffering from an unmet medical need.^10−12^

Recently, we have focused our attention on agents targeting the Wnt/β-catenin signaling pathway. Our early studies provided preclinical proof-of-concept for combining β-catenin and Na^+^/H^+^ exchanger 3 regulating factor 1 (NHERF1) pharmacological inhibitors as a novel strategy to enhance the apoptotic cell death of CRC resistant to current Wnt/β-catenin-targeted drugs.^13^ We reported new sulfonamide inhibitors of β-catenin signaling of potential therapeutic value as anticancer agents; compound **1** curbed on the Wnt reporter, reduced c-Myc levels, and prevented HCT116 colon cancer cell growth with submicromolar IC_50_ values.^14^ FH535 (**2**) is an inhibitor of the TCF/β-catenin signaling pathway that suppresses β-catenin/TCF-mediated transcription without affecting β-catenin levels^12^ and induces CRC cell cycle arrest without significant apoptosis.^15^ Sulfonamide **3**,^16^ in combination with NHERF1 inhibitors, prevented the growth of the CRC cells at submicromolar concentrations and showed higher effectiveness than combinations of the same NHERF1 inhibitors with **2**. MSAB (**4**) is an inhibitor of Wnt/β-catenin signaling with a selective antitumor effect on Wnt-dependent cancer cells in vitro and in mouse cancer models^17^ (Figure 1, panel A).

**Figure 1:**
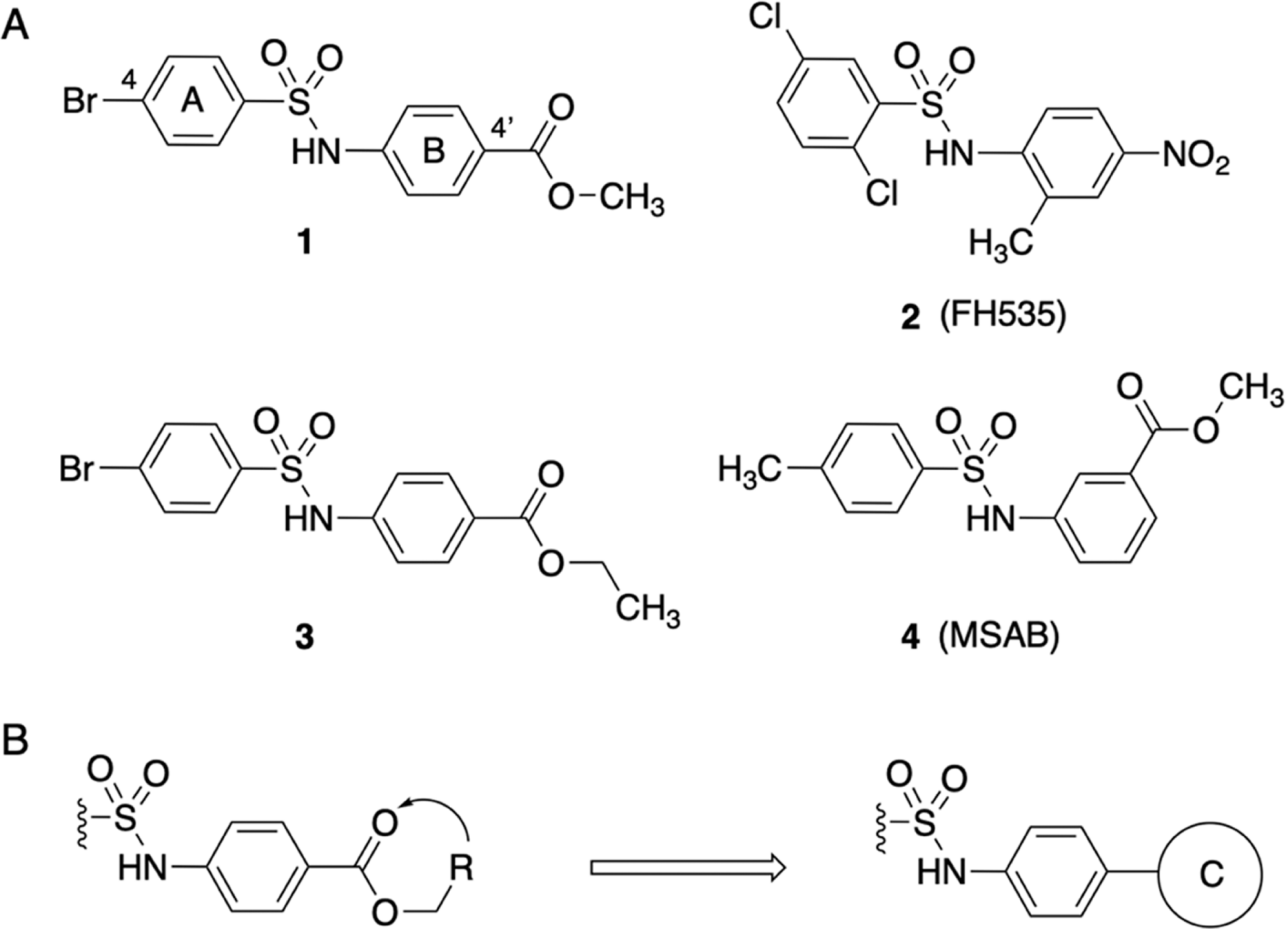
(A) Structures of compounds **1**–**4**. (B) Design of new β-catenin inhibitors **5**–**10**.

In a previous work, we successfully replaced the ester group with a five- or six-membered heterocyclic ring.^18−20^ Here, we decided to replace the ester function at ring B with a furan-2-yl or a pyridin-2-yl heterocyclic ring, keeping the bromine and chlorine atoms at position 4 of ring A (ring C, Figure 1, panel B). The new compounds were superior to the reference compound **3**(16) in reducing the luciferase activity and showed antitumor activity against the CRC SW480 and HCT116 cells (compound **9**) and triple-negative breast cancer (TNBC) MDA-MB-231, HCC1806, and HCC1937 cells (compounds **5** and **6**). Compound **9** shared the binding site with Tcf-4 (PDB ID 2GL7) within the hotspot binding region close to Lys508 of the β-catenin Armadillo repeats domain and abrogated the association between β-catenin and Tcf-4 in cells transfected with Myc-tagged Tcf-4. There is a substantial lack of structural data associated with the β-catenin inhibitors reported so far. For a number of inhibitors, the mechanism of action was inferred by the biological data;^21^ for others, just a putative binding area was proposed,^17^ and only for one derivative was reported an experimental binding mode.^22^ To our knowledge, **9** is the first small molecule ligand of this crucial region to be reported.

## Results and Discussion

### Chemistry

#### Synthesis of Compounds **5**–**10**

**Scheme 1:**
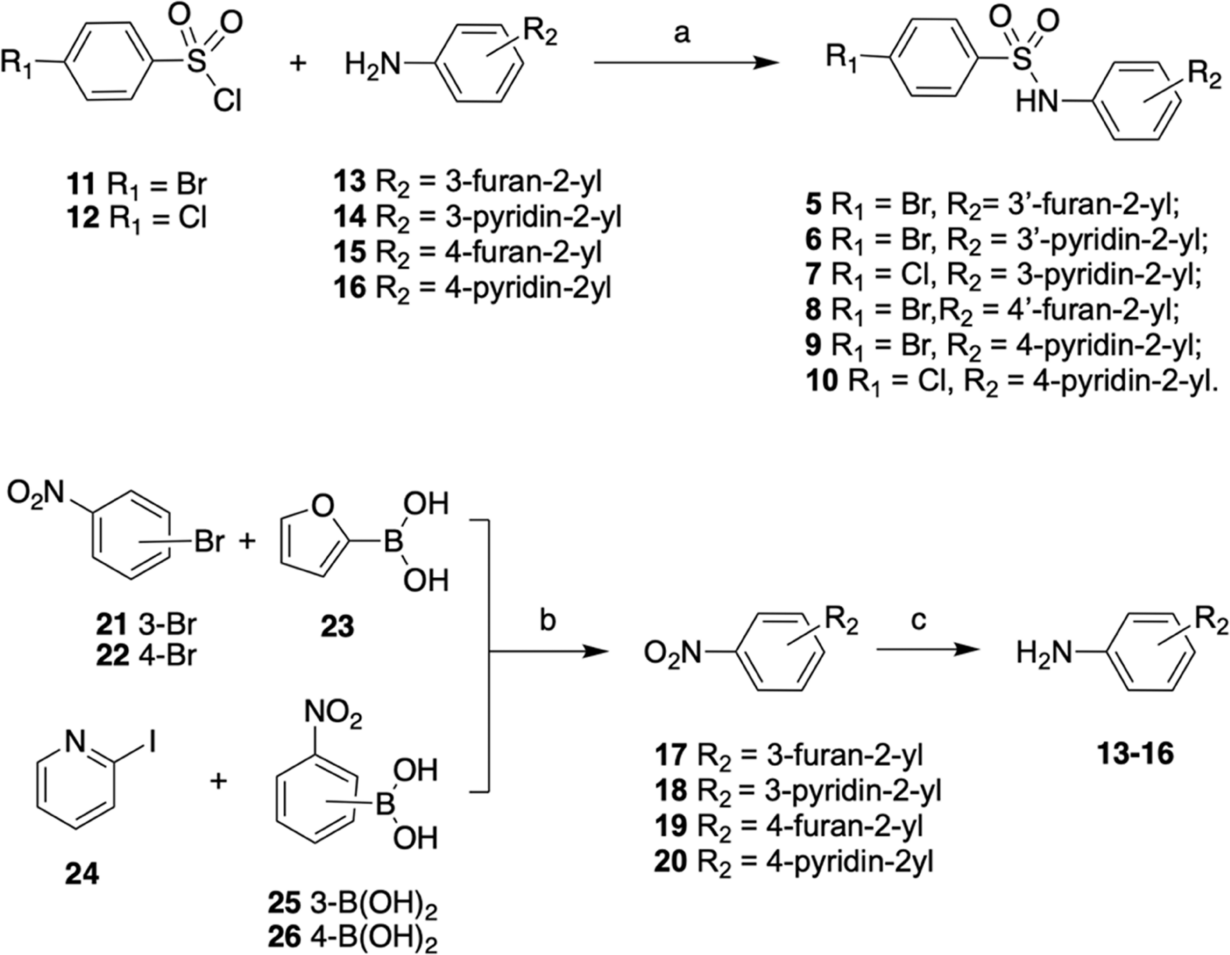
Reagents and Reaction Conditions (a) Dry pyridine, 120 °C, 2 h, Ar, 28–94%; (b) (i) Na_2_CO_3_, aqueous THF, degassed, 15 min, 25 °C; (ii) Pd(dppf)Cl_2_, reflux, 1.5 h, Ar, 30–80%; (c) Tin(II) chloride diihydrate, AcOEt, 80 °C, 3 h, 36–99%.

Compounds **5**–**10** were synthesized by a reaction of 4-bromobenzenesulfonyl chloride (**11**) or 4-chlorobenzenesulfonyl chloride (**12**) with 3-(furan-2-yl)aniline (**13**), 3-(pyridin-2-yl)aniline (**14**), or the corresponding 4-(furan-2-yl)- (**15**) and 4-(pyridin-2-yl)aniline (**16**) in dry pyridine at 120 °C for 2 h under an argon atmosphere. Anilines **13**–**16** were obtained by Tin(II) chloride diihydrate reduction of nitroderivatives **17**–**20** in ethyl acetate at 80 °C for 3 h. Nitroderivatives **17** and **19** were synthesized by a reaction of 3-bromo- (**21**) or 4-bromonitrobenzene (**22**) with furan-2-boronic (**23**) acid in the presence of sodium carbonate and [1,1′-bis(diphenylphosphino)ferrocene]dichloropalladium(II) in tetrahydrofuran at reflux for 1.5 h under an argon atmosphere. Similarly were prepared nitroderivatives **18** and **20** by a reaction of 2-iodopyridine (**24**) with 3-nitrophenyl- (**25**) or 4-nitrophenylboronic acid (**26**) (Scheme 1).

#### Crystallographic Studies

##### Binding of **9** in the β-Catenin Armadillo Repeats Domain

**Figure 2:**
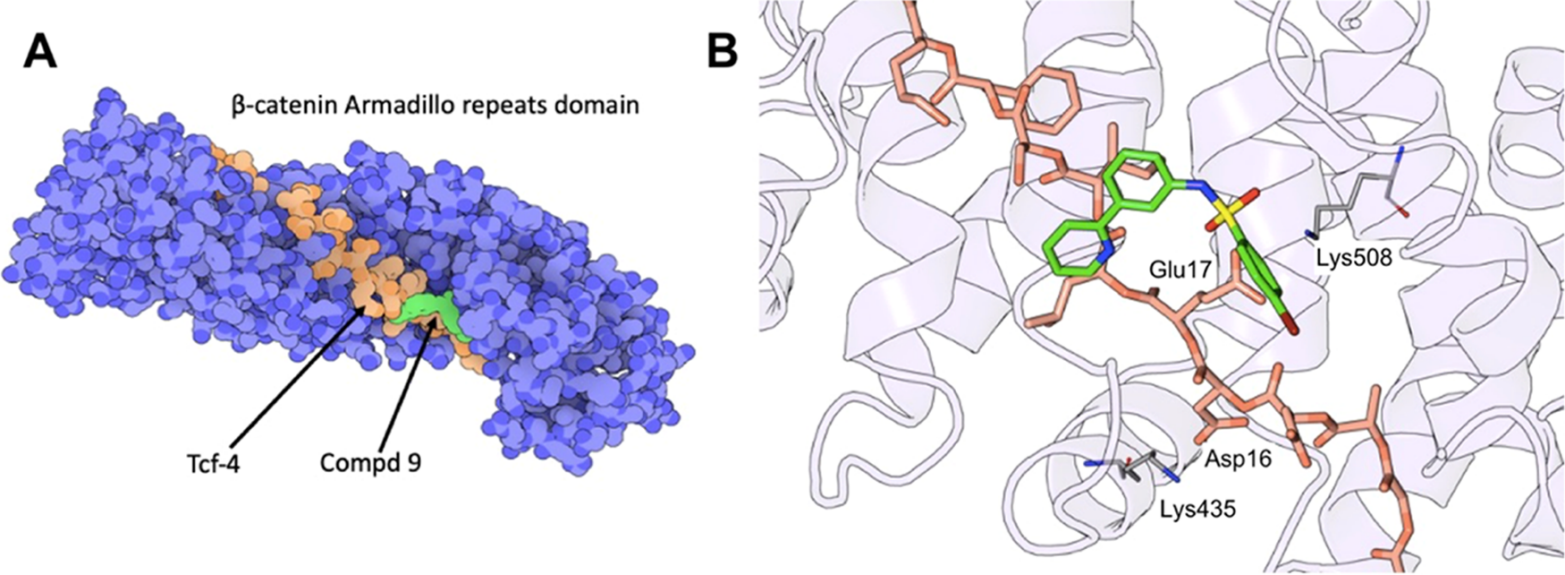
Crystal structure of **9** in a complex with the β-catenin Armadillo repeats domain. (A) Superposition of the β-catenin Armadillo repeats domain in a complex with Tcf-4 (orange; PDB ID 2GL7) and **9** (green; PDB ID 7ZRB). (B) Superposition of Tcf-4 and **9** within the “hotspot region 1” of the β-catenin Armadillo repeats domain. Figures were generated using the Protein Imager.^23^

**Figure 3:**
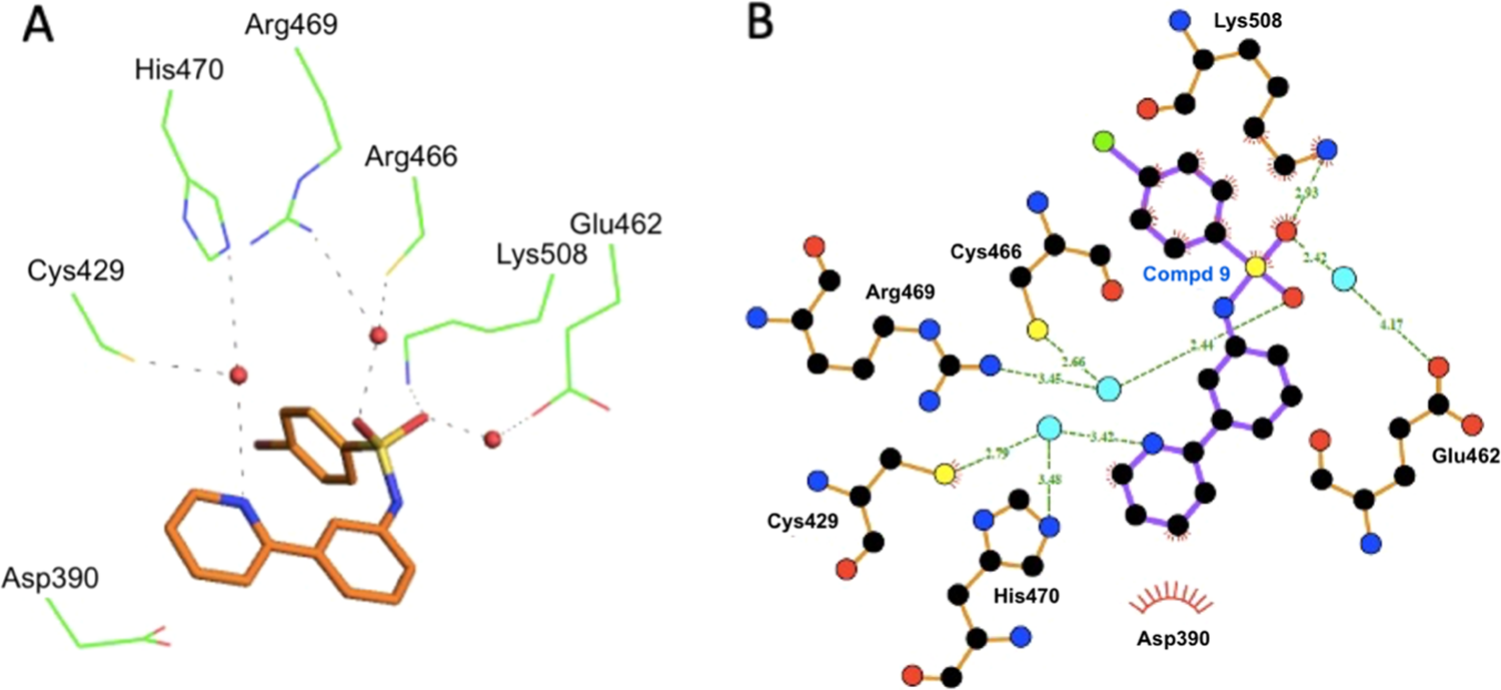
Summary of the interactions between **9** and the β-catenin Armadillo repeats domain. (A) Schematic drawing of the H-bond interactions. Structural water molecules are shown as red balls, and H-bonds are shown as dashed lines. (B) Two-dimensional schematic diagram of H-bonds and hydrophobic interactions generated by LigPlot. Structural water molecules are shown as cyan balls.

With the aim to elucidate the binding mode of **9** within the β-catenin Armadillo repeats domain, the crystal structure of the complex has been determined at 3.4 Å resolution from apo-protein crystals soaked with the ligand. The ligand, which can be easily accommodated into the final 2Fo-Fc electron density map (Figure S1, Supporting Information), occupies a solvent-exposed cavity resulting from the curvature of the α-solenoid superhelix involving the Armadillo repeats 7 to 10 (Figure 2A). As shown in Figure 3, the cationic side chain of Lys508 strongly interacts with the ligand through electrostatic interactions with one of its sulfonamide oxygens and with the cation-π attractive force of the bromophenyl aromatic ring. In addition, the heterocycle ring of **9** forms van der Waals contacts with the side chain of Asp390 and Cys429. Three structural water molecules actively contribute to coordinating the binding. In particular, a water molecule mediates H-bonds between the second sulfonamide oxygen with the sulfhydryl-reactive group of Cys466 and NH of Arg469. A second water molecule bridges the side chains of His470 and Cys429 with the nitrogen heterocycle ring of **9**. Finally, a third water links the first sulfonamide oxygen to the side chain of Glu462. Docking proposed binding modes of derivative **9** and reference compound **3** are depicted in the Supporting Information, Figure S2.

Interestingly, Lys508 has been previously described as a part of the “hotspot region 1” for the binding with the transcription factor Tcf-4^24,25^ along with Lys435. Indeed, these two positively charged residues are typically involved in salt-bridge hydrogen bonds with Glu17 and Asp16, respectively, playing a key role in Tcf-4 functional binding and the transcriptional activity of Wnt downstream genes. As depicted in Figure 2B, the superposition of the β-catenin Armadillo repeats domain with **9** and Tcf-4 (PDB ID 2GL7) demonstrates that the two ligands share a common binding site within the hotspot binding region close Lys508. Actually, **9** represents the first small molecule to be described in such a crucial region of this domain, which is still deemed by most as an “undruggable” target.^22,26^

#### Surface Plasmon Resonance

Surface plasmon resonance biosensor technology was used to directly measure the binding affinity of **9** to the β-catenin Armadillo repeats domain. To collect detailed kinetic data of the binding measured as the rate constants of the association (*k*_a_) and dissociation (*k*_d_) of the complex, a concentration series of the analyte ranging from 1.95 up to 62.5 nM was injected over the covalently immobilized Armadillo repeats domain onto the sensor chip surface and the interaction between the analyte and the ligand was detected as a measure of the change in mass concentration upon the surface, expressed in resonance units (RUs) and compared to the baseline.

**Figure 4:**
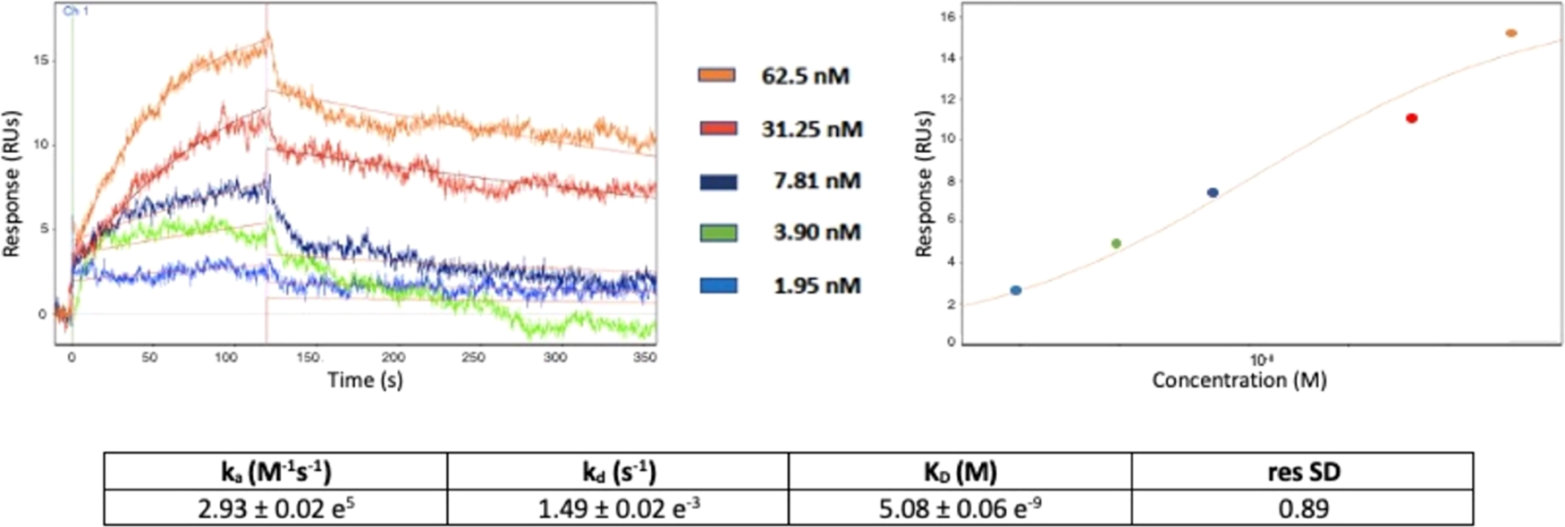
SPR kinetic analysis. The sensorgrams (left panel) and dose–response plot (right panel) of **9** binding to the β-catenin Armadillo repeats domain at different concentrations, with the determined binding parameters listed in the bottom table.

Full kinetic analysis of the binding interaction between **9** when binding to the Armadillo domain is shown in Figure 4, with the calculated binding parameters listed in the bottom table. As evidenced by the sensorgram obtained at different analyte concentrations, the dissociation rate constant of **9** is very slow, thereby resulting in a low nanomolar equilibrium affinity constant (*K*_D_) of the binding calculated as the ratio of *k*_d_/*k*_a_ (Figure 4).

### Biology

#### Inhibition of Luciferase Activity

**Figure 5:**
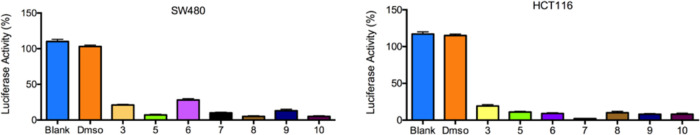
Residual luciferase activity (%) by compounds **5**–**10** and reference compound **3** in SW480 cells (left panel) and HCT116 cells (right panel). Data are represented as the mean ± SD of three independent experiments, each performed in triplicate.

We synthesized derivatives **5**–**10** bearing 5-membered and 6-membered heterocyclic rings at the position 3′ or 4′ of the B phenyl ring. The compounds were assayed in the Topflash/Foplash luciferase report assay in SW480 and HTC116 cells at 30 μM concentration to measure the activity of the Wnt/β-catenin signaling pathway according to the previously reported literature.^27^ In SW480 and HCT116 cells, β-catenin is localized predominantly in the nucleus and plasma membrane. SW480 cells show higher Wnt activation compared to HCT116 cells.^28^ In this assay, all compounds, except **6** in SW480 cells, strongly reduced the luciferase activity and were superior to the previously reported compound **3**.^15^ In SW480 cells, compounds **5**–**10** were in the 1.75%(**10**)–7.32%(**7**) range of residual luciferase activity, except **6**(28.46%), compared to **3**(21.61%); in HCT116 cells, compounds **5**–**10** were in the 0.37%(**7**)–5.88%(**5**) range, compared to **3** (18.75%) (Table 1 and Figure 5).

**Table 1:**
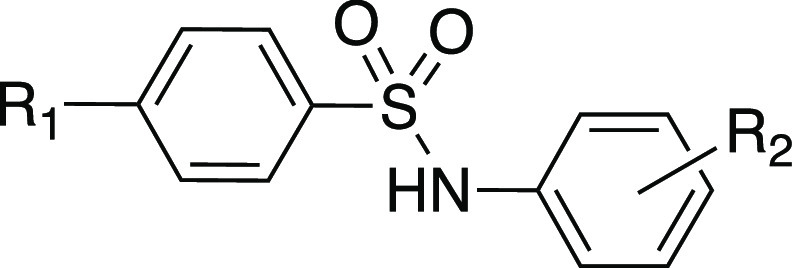

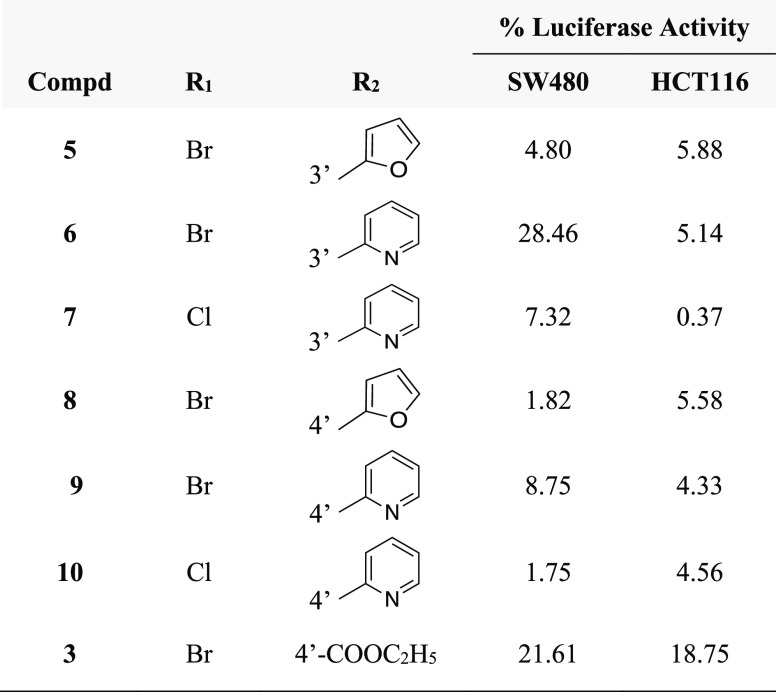
Residual Luciferase Activity (%) by Compounds **5**–**10** and Reference Compound **3**^a^

a Experiments were performed in duplicate or triplicate.

**Figure 6:**
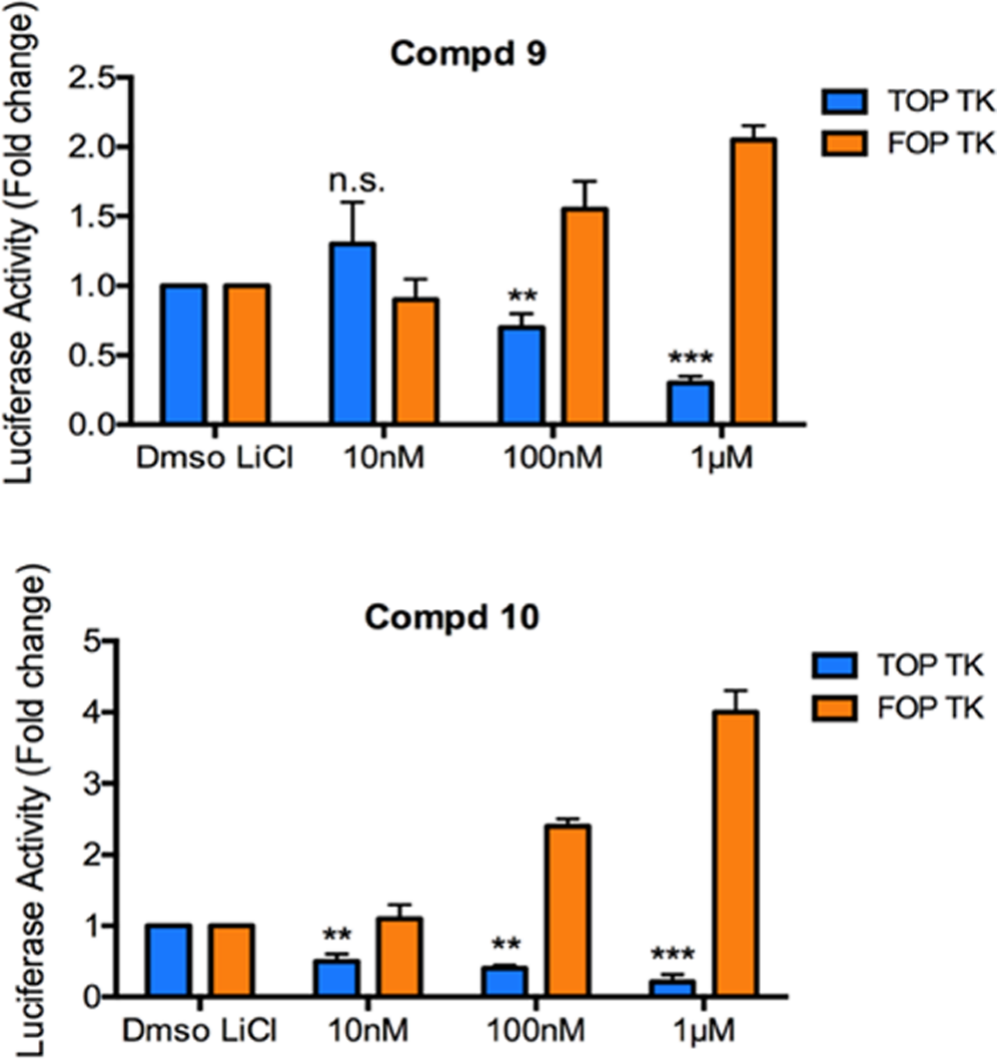
HCT116 cells were transfected with luciferase-based vectors and treated with LiCl (50 mM) together with increasing concentrations of compound **9** or **10**. Cells were harvested 24 h post-treatment and assayed for luciferase activity. Inhibitions were calculated as the luciferase/renilla ratio of the treated samples vs. the luciferase/renilla ratio of the untreated (control) samples. Data are represented as the mean ± SD of three independent experiments, each performed in triplicate. **p < 0.01, ***p < 0.001, n.s = not significant, as determined by analysis of variance (ANOVA).

Compounds **9** and **10** were assayed at increasing 10 and 100 nM and 1 μM concentrations in HCT116 cells transfected with luciferase-based vectors and treated with LiCl (50 mM). TOP TK is a well-established Wnt reporter vector containing eight sequences of Tcf/Lef binding site upstream of the timidine kinase (TK) minimal promoter, driving the expression of firefly luciferase. In the FOF TK plasmid, the Tcf/Lef binding sites contain mutated sequences, and the reporter does not respond to Wnt activation. Compounds **9** and **10** inhibited β-catenin with IC_50_ values of 6.3 and 8.2 μM, respectively, and were more effective than reference compound **3** (IC_50_ = 14.1 μM, lit.^14^) (Figure 6).

#### Inhibition of Myc Expression

**Figure 7:**
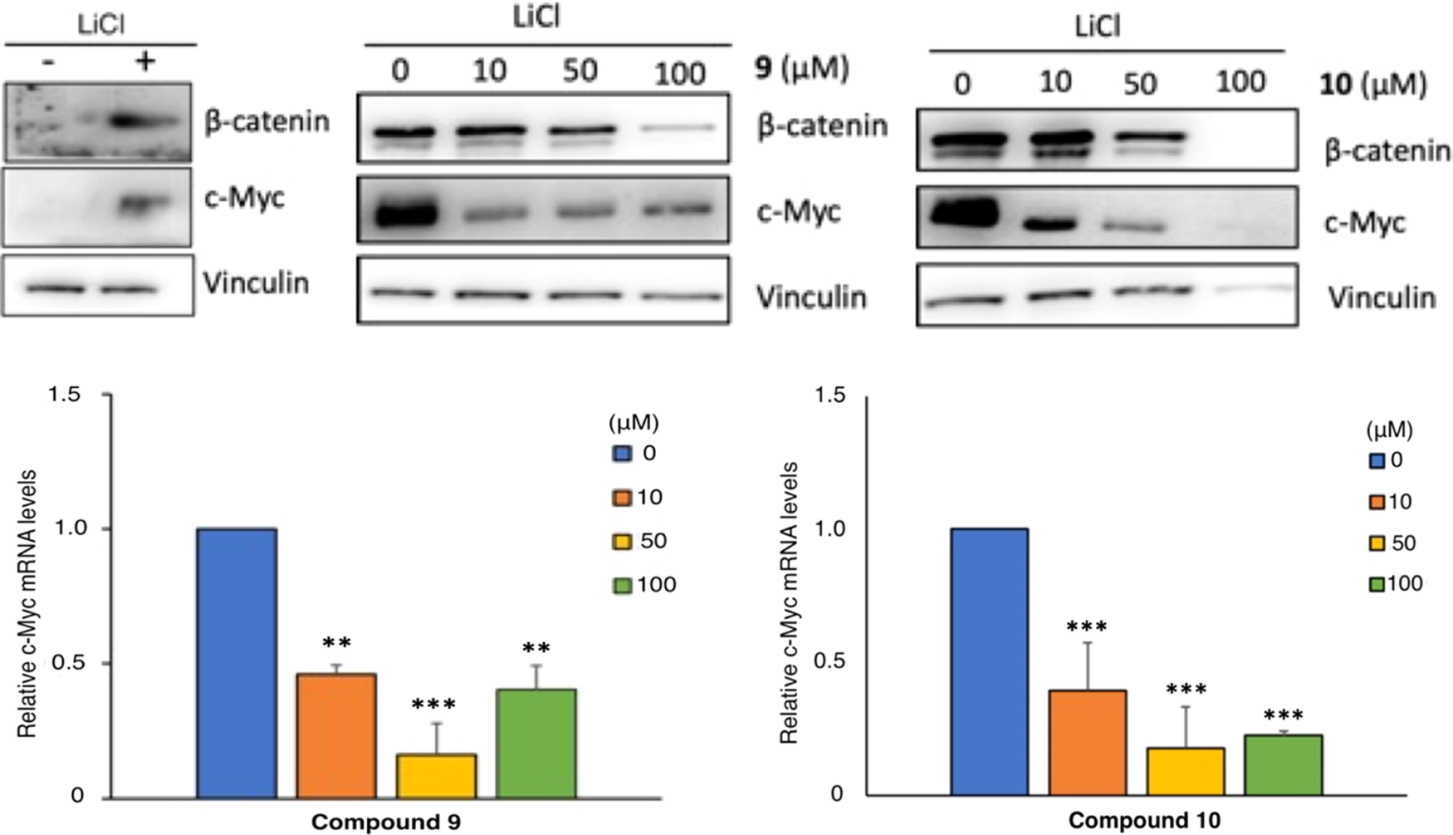
Top panels. HCT116 cells were treated with LiCl (50 mM) and with compounds **9** or **10** at the indicated concentrations for 24 h. β-catenin and c-Myc levels were analyzed by western blot. Vinculin was used as a loading control. The left control panel shows the increase of MYC protein levels in response to LiCl treatment. Bottom panels. c-Myc mRNA levels were measured by qPCR and normalized to the expression of β-actin mRNA and expressed as fold change relative to the control sample. Results represent the mean ± SD of three independent experiments, each performed in triplicate. **p < 0.01, ***p < 0.001 as determined by ANOVA.

**Figure 8:**
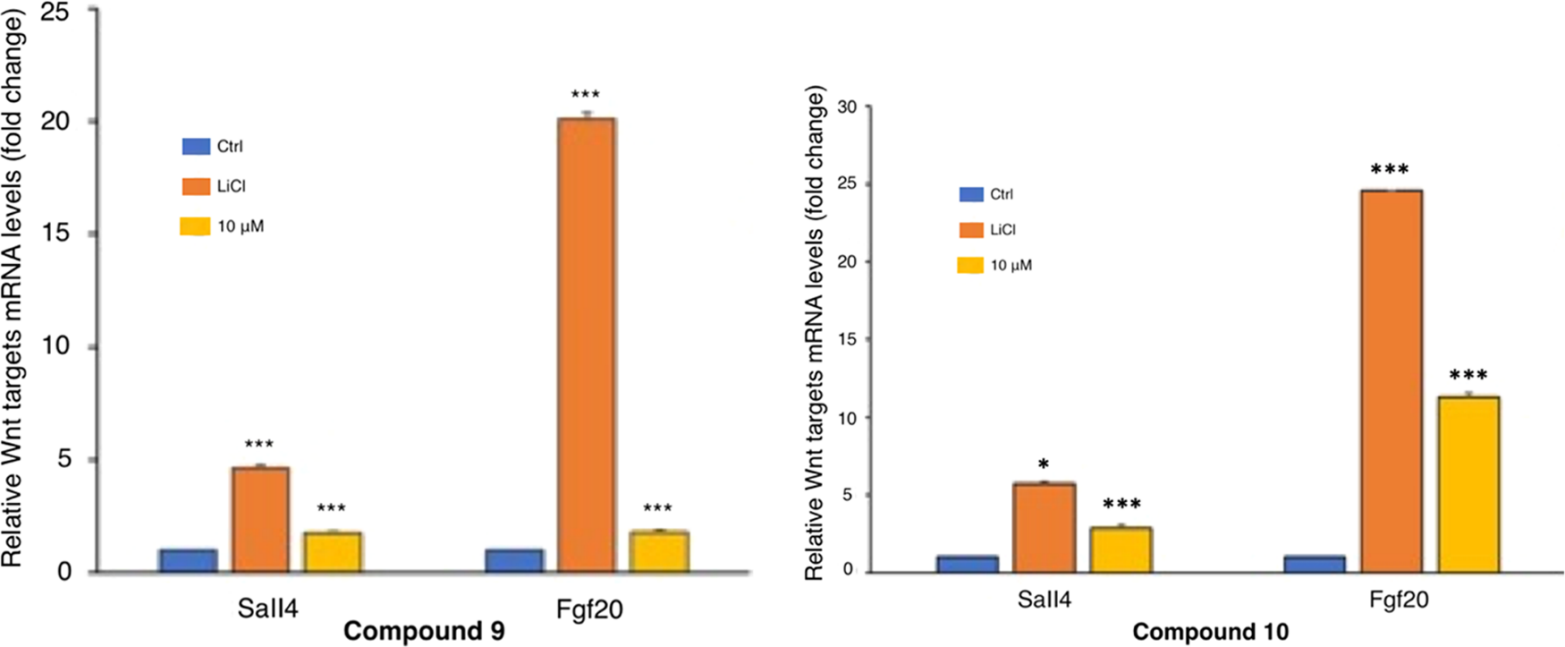
Compounds specifically inhibit Wnt-target genes in HCT116 colon cancer cells. HCT116 was treated with LiCl (50 mM) and 10 μM compound 9 or **10** for 24 h. Fgf20, Sall4 (Wnt-target genes) mRNA levels were measured by qPCR and normalized to the expression of β-actin mRNA. Results are expressed as fold change relative to the control sample and represent the mean ± SD of three independent experiments, each performed in triplicate. *p < 0.05, **p < 0.01, ***p < 0.001 as determined by ANOVA.

We next tested the effect of compounds **9** and **10** on the expression of the known Wnt/β-catenin target gene Myc. To this end, HCT116 cells were treated for 24 h with 50 mM LiCl and increasing concentrations (10–50–100 μM) of the two compounds. As shown in Figure 7, both compounds induced a significant dose-dependent inhibition of both proteins and mRNA levels of Myc, while β-catenin protein levels were reduced only at the highest dose of both compounds, likely reflecting a certain degree of toxicity of the two reagents at those concentrations, as suggested by the parallel decrease of Vinculin at the same dose (Figure 7). The inhibition of Myc expression was significantly robust (about 2.5-fold inhibition) at doses as low as 10 μM. At the same concentration, the mRNA levels of two additional Wnt-target genes, Fgf20 and Sal4,^13^ were strongly inhibited by both compounds **9** and **10** (Figure 8). Hence, these data suggested that the two compounds may impair one of the activating steps occurring after β-catenin stabilization.

#### Inhibition of the β-Catenin/Tcf-4 Interaction

**Figure 9:**
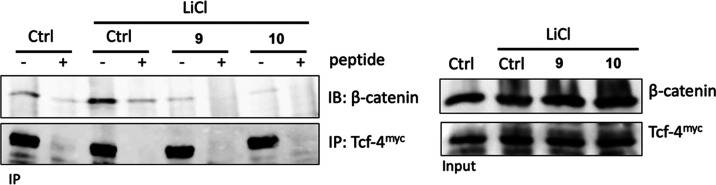
Co-immunoprecipitation assay of β-catenin and the Myc-tagged Tcf-4 protein. HCT116 cells were transiently transfected with the Myc-tagged Tcf-4 plasmid and treated with LiCl (50 mM) and **9** (50 μM) or **10** (50 μM) for 24 h. Lysates were immunoprecipitated using Myc-agarose beads and western blot analysis was performed. Co-immunoprecipitated endogenous β-catenin protein levels are shown (IP panel), as well as the expression levels of Tcf-4 and β-catenin (Input panel). Myc-agarose beads saturated with the Myc-blocking peptide before immunoprecipitation were used as a negative control.

Based on the prediction gathered from the crystallographic studies, we addressed the possibility that compounds **9** and **10** might impair the association between β-catenin and Tcf-4. Toward this end, we performed a co-immunoprecipitation study in HCT116 cells transfected with Myc-tagged Tcf-4, treated with the two compounds, and immunoprecipitated with Myc antisera. As shown in Figure 9, the endogenous β-catenin protein was associated with ectopic Tcf-4, and the binding was increased upon the addition of LiCl, as expected. The association was completed by the Myc-blocking peptide, documenting the specificity of the binding between β-catenin and Tcf-4. Notably, exposure to 50 μM compound **9** or **10** completely abrogated the association, thus validating the prediction of crystallographic studies that both compounds interact with sites of the β-catenin Armadillo repeats domain that are indispensable for the association between the two proteins. Together, these results demonstrate that both compounds **9** and **10** significantly inhibit Wnt-dependent target gene expression by preventing the β-catenin/Tcf-4 interaction.

#### Inhibition of Cancer Cell Growth: SW480 and HCT116 Cells

The SW480 colon carcinoma cells are constitutively active for β-catenin/Tcf transcriptional activity. The SW480 cells carry a mutation in the APC tumor suppressor gene product, resulting in sustained upregulation of the β-catenin/TCF signaling pathway and expressing low amounts of E-cadherin.^29,30^ Similarly, HCT116 cells, with a mutation in the CTNNB1 gene that encodes β-catenin, present a well-enhanced Wnt/β-catenin pathway.^31^ Compounds **5**–**10** inhibited the SW480 and HCT116 colon cancer cells with EC_50_ values at micromolar concentrations. Compounds **5**, **6**, and **8**–**10** inhibited the SW480 cell with similar inhibitory potency; the EC_50_ values went from 27 μM (**6**) to 37 μM (**10**). Compounds **9** and **10** with EC_50_ values of 24 and 27 μM, respectively, were the most potent inhibitors of the HCT116 colon cancer cells. Compared with 5-FU, a reference drug for the treatment of colon cancer, compound **9** yielded a 6-fold improvement in the SW480 cell growth inhibition, while 5-FU alone strongly inhibited the HCT116 cells with EC_50_ of 8 μM. The activities of 5-FU were not surprising since HCT116 cells were identified to be the most sensitive cells, SW480 were the least sensitive, and SW620 cells showed intermediate sensitivity.^29^ In SW480 cells, a combination treatment of 5-FU and **9** (EC_50_ = 63 μM) showed superior efficacy than a single treatment with 5-FU (EC_50_ = 217 μM) but less efficacy than a single treatment with **9** (EC_50_ = 34 μM), suggesting that 5-FU may, at least partially, diminish the **9**-induced cytotoxicity. Similarly, in SW620 cells, the **9** + 5-FU combination treatment yielded EC_50_ = 46 μM compared to **9** or 5-FU alone with EC_50_ of 37 and 103 μM, respectively. On the contrary, compound **9** did not show a synergistic effect with 5-FU. The different sensitivity of the SW480 and HCT116 cells to 5-FU has been correlated to the activation of protein kinase C type δ (PKCδ) and caspase 9 in HCT116 cells, but not in the SW480 cells.^32^ PKCδ has been recognized to be a proapoptotic kinase; its cleavage and activation promote apoptotic cell death (Table 2).^33^

**Table 2:** In Vitro Inhibitory Activity of SW480 and HCT116 Colon Cancer Cells by Compounds **5–10** and 5-FU as the Reference Compound^a^

	EC_50_ (μM)^b^	
compd	SW480	HCT116
**5**	28	50
**6**	27	45
**7**	>50	>50
**8**	45	47
**9**^c^	34	24
**10**	37	27
5-FU^d^,^e^	217	8
**9**^f^,^g^ + 5-FU	63	8

a Experiments were performed in duplicate or triplicate.

b SD: Standard deviations went from ± 5% to ± 10% of the indicated EC_50_ values.

c Inhibition of SW620 cells: **9**, EC_50_ = 37 μM.

d 5-FU, 5-fluorouracil.

e Inhibition of SW620 cells: 5-FU, EC_50_ = 103 μM.

f Inhibition of SW620 cells: **9**^g^ + 5-FU, EC_50_ = 46 μM.

g **9** was at a constant concentration of 0.25 μM.

#### MDA-MB-231 Cells, Triple-Negative Breast Cancer Adenocarcinoma Cell Line, Mesenchymal Subtype

TNBC represents approximately 10–15% of all breast cancers and is the most aggressive subtype of breast cancer. Patients with TNBC have a poor outcome compared to the other subtypes of breast cancer. In the breast cancer tissue, β-catenin is overexpressed compared to the normal tissue. The overexpression of β-catenin is associated with TNBC and lymph node metastasis. TNBC does not present the expression of the markers estrogen receptor (ER) and progesterone receptor (PR) and amplification of HER-2/Neu.^34−36^ There is a quest for the development of new therapies to treat TNBC. Compounds **5–10** were assayed at scalar concentrations of 40, 13.33, 4.44, 1.48, and 0.49 μM in parallel with the reference compounds **3**, ICG-001, an inhibitor of TCF/β-catenin-mediated transcription, and iCRT3, a selective cell-permeable β-catenin responsive transcription (CRT) inhibitor (Figures S3–S11, Supporting Information). Effects on cell proliferation of MDA-MB-231 TNBC cells were evaluated by live-time cell imaging using the Incucyte S5 system. These analyses revealed that compounds **5** (Figure S4) and **6** (Figure S5) were the most effective among the novel tested compounds **5–10**, displaying significant antiproliferative effects in MDA-MB-231 cells already at 48 and 72 h at the concentration of 40 μM. Compared to iCRT3 (Figure S10), compounds **5** (Figure S4) and **6** (Figure S5) showed stronger effects, achieved at slightly earlier time points and at higher doses. ICG-001 was the most effective compound in reducing MDA-MB-231 cell viability even at the low dose of 0.49 μM (Figure S11).

#### HCC1806 and HCC1937 cells, Triple-Negative Breast Cancer Adenocarcinoma Cell Lines, Basal Subtype

**Figure 10:**
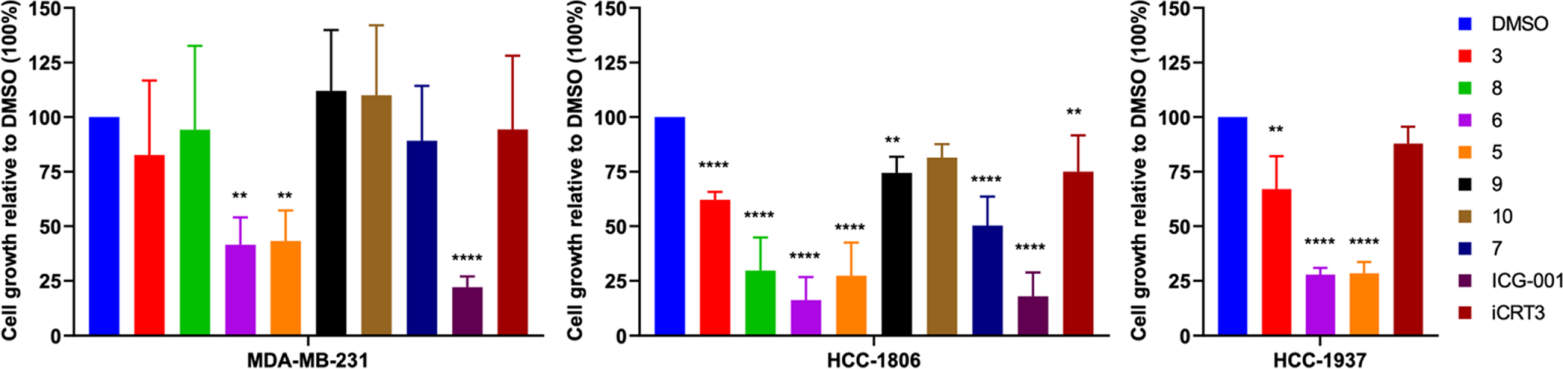
Inhibition of MDA-MB-231, HCC1806, and HCC1937 TNBC cell growth by compounds **5**–**10** and reference compounds **3**, IGC-001, and iCRT3 at 40 μM. Cell growth inhibition was quantified using the IncuCyte cell-by-cell analysis software, by comparison with the growth of DMSO-treated cells at 72 h, set at 100% (mean ± SD, *n* = 3, one-way ANOVA, **p ≤ 0.01, ****p ≤ 0.0001).

**Figure 11:**
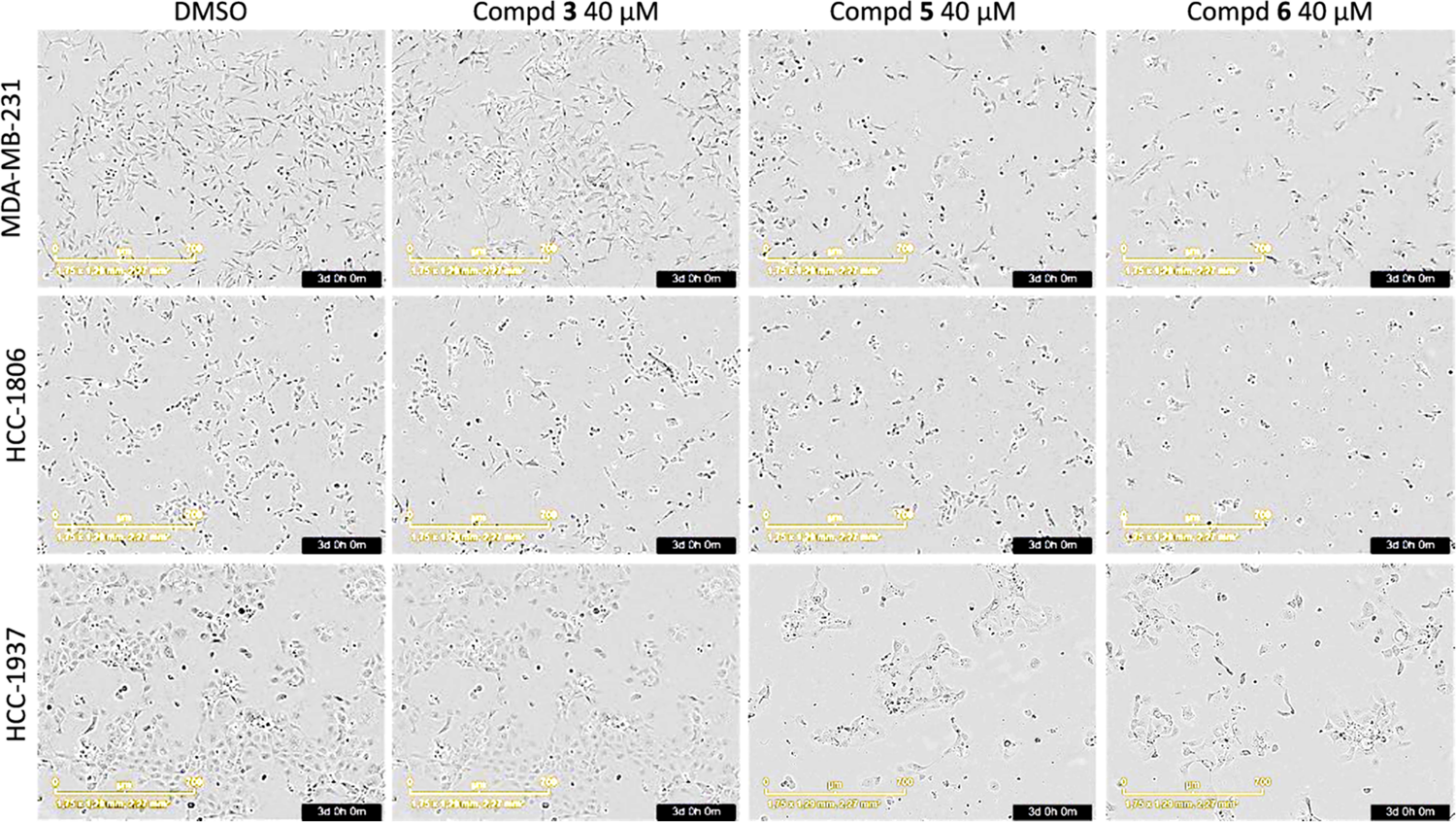
Representative images of MDA-MB-231, HCC1806, and HCC1937 TNBC cells at 3 days post-treatment with compounds **1**, **5**, and **6** at 40 μM. Images were taken using the live-time cell imaging system IncuCyte.

As TNBC is a highly heterogeneous subgroup, including breast cancers with different molecular and phenotypic traits,^37^ effects of compounds **5–10** on cell proliferation were also tested in additional TNBC cell lines, i.e., HCC1806 and HCC1937. As inhibitors of HCC1806 TNBC cells, compounds **5–10** were evaluated in parallel with compounds **3**, ICG-001, and iCRT3 (Figures 10 and S12–S19, Supporting Information); as inhibitors of the HCC1937 TNBC cells, **5–10** were compared with **3** and iCRT3 (Figures 10, S20, and S21). Effects on cell proliferation were evaluated as above for the MDA-MB-231 TNBC cells at the latest time point of observation of 72 h. Collectively, compounds **5** and **6** displayed the highest antiproliferative effect in multiple TNBC cell lines (Figure 10). Compounds **7** and **8** showed a mild inhibitory effect toward HCC1806 TNBC cell growth (no data against HCC1937 TNBC cells) (Figures 10, S13, and S14, Supporting Information). Reference compound **3** was ineffective in reducing MDA-MB-231 TNBC cell growth and showed mild efficacy against HCC1806 and HCC1937 TNBC cells (Figures 10, 11, and S3–S21, Supporting Information).

#### In Vivo Tumor Growth Inhibition by Compound **9**

**Figure 12:**
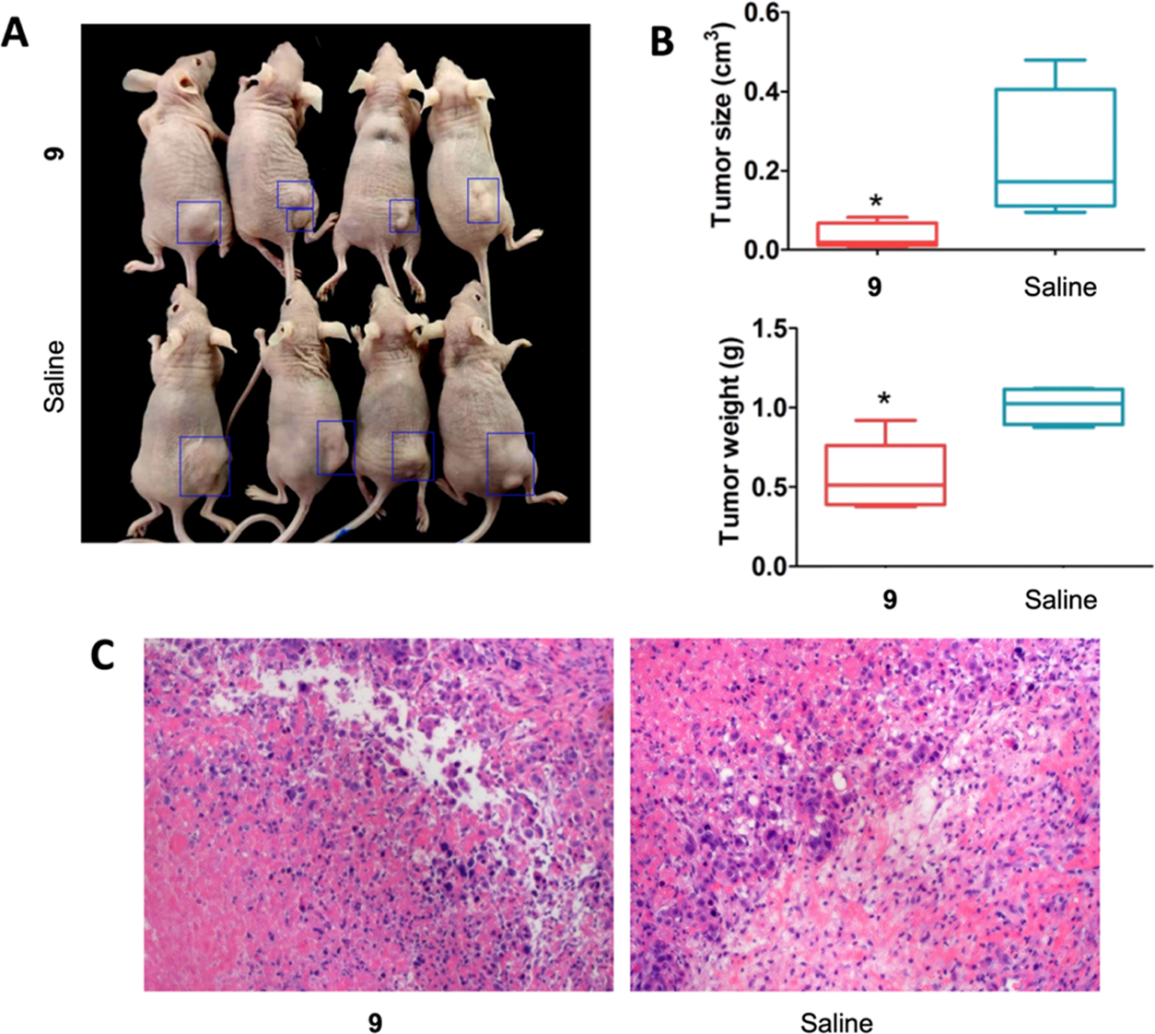
Compound **9** inhibited the in vivo tumorigenicity of human colorectal cancer line HCT116. (A) Tumor tissues. (B) Top panel. The volume of tumors originating. *p < 0.05 vs. the control group; *t*-test; *n* = 4. (B) Bottom panel. The weight of tumors originating. *p < 0.05 vs. the control group; *t*-test; *n* = 4. (C) HE staining revealed that two group tumors were human colorectal cancer. Magnification 200×.

Ten-week-old female BALB/C^nu/nu^ mice were inoculated subcutaneously with HCT116 cells (1 × 10^8^ cells/mL) at the logarithmic growth phase. Compound **9** (100 μL, 25 mg/kg) was administered through an intraperitoneal injection every 2 days after tumorigenesis. Mice were euthanized on day 30, and tumors on the backs were collected for the measurement of tumor volume and weight. Results showed that the tumors from the group treated with compound **9** were significantly smaller than those formed from the control group (saline treatment) in terms of both tumor volume and weight (Figure 12A,B). Haematoxylin and eosin (HE) staining shows that the type of tumor formed in the two groups was colorectal cancer (Figure 12C).

**Figure 13:**
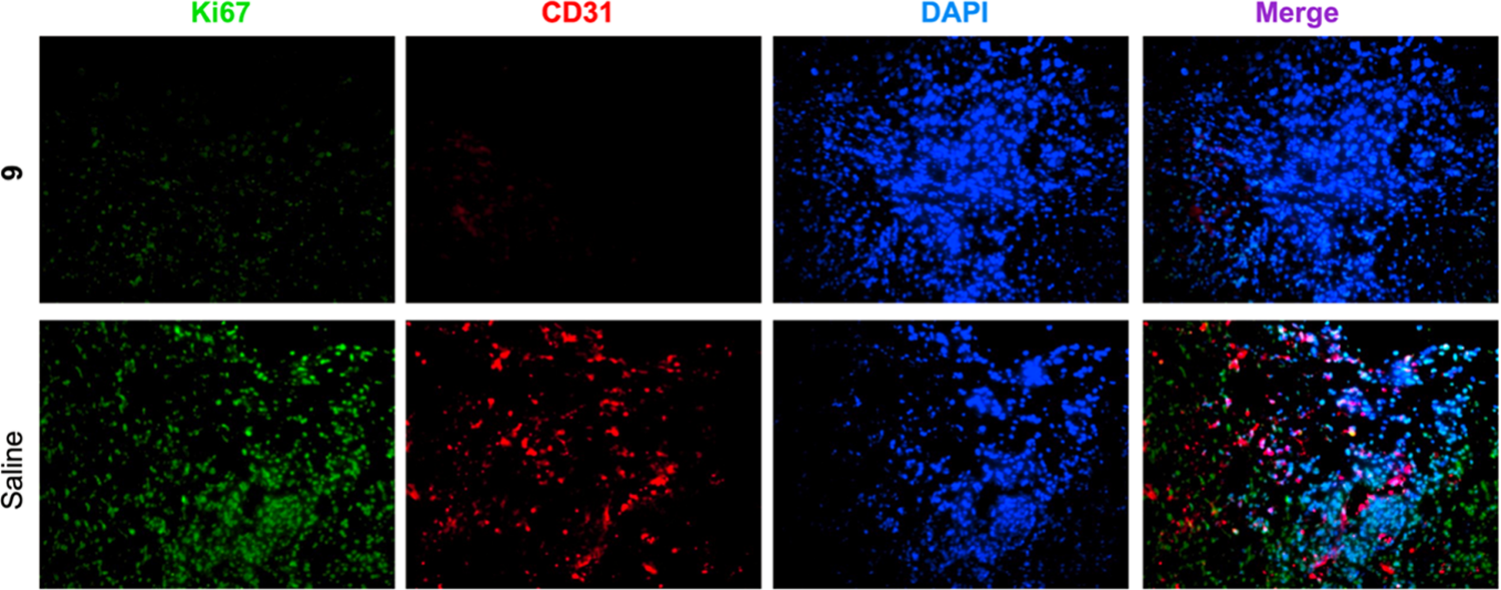
Immunofluorescence staining results of tumor tissues derived from the group treated with compound **9**. Magnification 200×.

Immunofluorescence staining results showed that the tumor tissues derived from the group treated with compound **9** have a significantly lower expression of the proliferation factor Ki67 and the angiogenesis marker CD31 (Figure 13). Nuclear protein Ki67 is a proliferation marker of tumor cell proliferation and is an indicator in cancer diagnosis through a biopsy.^38^ CD31 is a marker of angiogenesis, along with the vascular endothelial growth factor (VEGF).^39^ CD31 has been found in the angiogenetic early breast cancer tissues and is highly expressed on the surface of endothelial cells.^40^ Thus, our results suggested that **9** inhibits in vivo proliferation of cancer cells.

#### Drug-like Properties

**Figure 14:**
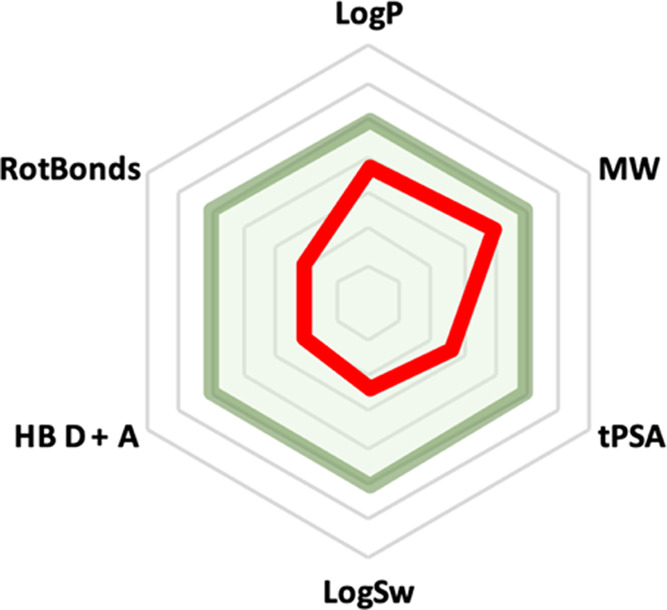
Radar plot of drug-like properties of compound **9**. The light green colored zone represents the suitable physicochemical space for oral bioavailability. −1 < Log *P* < 5; 150 < *M*_W_ < 500; 20 Å^2^ < tPSA < 130 Å^2^; −10 < Log Sw < 0; 0 < HBD + A < 10; 0 < RotBonds < 9. The red line represents values for compound **9**.

The ADME profile of **9** was predicted by SwissADME website^41^ representative descriptors (Table 3). Compound **9** does not violate the Lipinski^42^ and Veber^43^ rules, and through computed Caco-2 permeability, shows good bioavailability and capability to cross the gut/blood barrier (Table 3 and Figure 14).

**Table 3:** Compound **9** ADME Profile

comp	Log* P*^a^	*M*_W_^b^	Log Sw^c^	tPSA^d^	HBA^e^	HBD^f^	Rot^g^	CACO^h^
**9**	3.73	389.27	–4.92	67.44	3	1	4	1015

a Logarithm of the partition coefficient between *n*-octanol and water computed by the XLOGP3 method.

b Molecular weight.

c Log Sw represents the logarithm of compound water solubility computed by the ESOL method. Log Sw predicted compound aqueous solubility values: > −10: insoluble, > −6: poorly soluble, > −4: moderately soluble, > −2: soluble, > 0: high soluble.

d Molecular polar surface area, this parameter has been shown to correlate with human intestinal absorption (<140).

e Number H-bond acceptors.

f Number H-bond donors.

g Number of rotatable bonds.

h Predicted apparent Caco-2 cell permeability in nm/sec. <25 poor, >500 great nm/s.

## Conclusions

We synthesized new N-(heterocyclylphenyl)benzensulfonamides derivatives as inhibitors of the β-catenin signaling pathway with a robust interaction with β-catenin. In the luciferase reporter assay in SW480 and HCT116 cells, compounds **5**–**10**, except **6** in SW480 cells, strongly reduced the luciferase activity and were superior to the previously reported reference compound **3**. Compound **9** was the most potent inhibitor of the HCT116 cells (EC_50_ = 24 μM) and showed consistent inhibition of the SW480 cells (EC_50_ = 34 μM). Compound **9** was chosen for the crystallographic studies. The crystal structure of compound **9** in a complex with the β-catenin Armadillo repeats domain was determined at 3.4 Å resolution from apo-protein crystals soaked with the ligand. Compound **9** occupied a solvent-exposed cavity resulting from the curvature of the α-solenoid superhelix involving the Armadillo repeats 7 to 10. Superposition of the β-catenin Armadillo repeats domain with **9** and Tcf-4 (PDB ID 2GL7) demonstrates that the two ligands share a common binding site within the hotspot binding region close to Lys508. Compound **9** represents the first small molecule to be described in such a crucial region of this domain. The binding affinity of **9** to the β-catenin Armadillo repeats domain was measured by surface plasmon resonance biosensor technology. The very slow dissociation rate constant of **9** accounted for a low nanomolar binding affinity constant and correlated with the hydrophobic and polar characteristics of this molecule.^44,45^ Most importantly, in a co-immunoprecipitation study, in cells transfected with Myc-tagged Tcf-4, exposure to compounds **9** or **10** at 50 μM completely abrogated the association between β-catenin and Tcf-4, thus validating the prediction of the crystallographic studies. The results demonstrate that both compounds **9** and **10** significantly inhibit Wnt-dependent target gene expression by preventing the β-catenin/Tcf-4 interaction. Compounds **5** and **6** were the most effective inhibitors of the MDA-MB-231 cells (TNBC adenocarcinoma cell line, mesenchymal subtype) and HCC1806 and HCC1937 cells (TNBC adenocarcinoma cell lines, basal subtype), displaying significant antiproliferative effects already at 48 and 72 h at the concentrations of 40 μM, while **9** was less effective against these cell lines. In mice, compound **9** reduced both tumor volume and weight of human colorectal cancer line HCT116, with a significantly lower expression of the proliferation factor Ki67 and the angiogenesis marker CD31. Compound **9** does not violate the Lipinski and Veber rules, and through computed Caco-2 permeability, shows good bioavailability and capability to cross the gut/blood barrier.

In summary, we described the synthesis and antitumor activities of novel *N*-(heterocyclylphenyl)benzensulfonamides β-catenin inhibitors. Crystallographic studies revealed that **9** binds to a previously unreported bind site of β-catenin abrogating the association between β-catenin and Tcf-4. Compound **9** induced in vitro cell death in SW480 and HCT116 cells and in vivo tumorigenicity of human colorectal cancer line HCT116. Two compounds, **5** and **6**, showed significant inhibition of the MDA-MB-231, HCC1806, and HCC1937 TNBC cells. These findings highlight the potential of this novel class of β-catenin inhibitors as anticancer agents and pave the way for further development. The results of further studies will be reported in a forthcoming publication.

## Experimental Section

### Chemistry

All reagents and solvents were handled according to the material safety data sheet of the supplier and were used as purchased without further purification. Organic solutions were dried over anhydrous sodium sulfate. Evaporation of solvents was carried out on a Büchi Rotavapor R-210 equipped with a Büchi V-850 vacuum controller and a Büchi V-700 vacuum pump. Column chromatography was performed on columns packed with silica gel from Macherey–Nagel (70–230 mesh). Silica gel thin-layer chromatography (TLC) cards from Macherey–Nagel (silica gel precoated aluminum cards with a fluorescence indicator visualizable at 254 nm) were used for TLC. Developed plates were visualized with a Spectroline ENF 260C/FE ultraviolet (UV) apparatus. Melting points (mp) were determined on a Stuart Scientific SMP1 apparatus and are uncorrected. Infrared (IR) spectra were recorded on a PerkinElmer Spectrum 100 Fourier transform infrared (FT-IR) spectrophotometer equipped with a universal attenuated total reflectance accessory and IR data were acquired and processed by PerkinElmer Spectrum 10.03.00.0069 software. The band position and absorption ranges are given in cm^–1^. Proton nuclear magnetic resonance (^1^H NMR) spectra were recorded with a Bruker Avance (400 MHz) spectrometer in the indicated solvent, and the corresponding fid files were processed by MestreLab Research SL MestreReNova 6.2.1-769 software. Carbon-13 nuclear magnetic resonance (^13^C NMR) spectra were recorded with a Bruker Avance (100 MHz) or a Bruker Avance Neo (176 MHz) spectrometer in the indicated solvent, and the corresponding fid files were processed by MestreLab Research SL MestreReNova 6.2.1e769 software. Chemical shifts of ^1^H and ^13^C NMR are expressed in δ units (ppm) from tetramethylsilane. Ethyl 4-((4-bromophenyl)sulfonamido)benzoate (**3**) was synthesized as we previously reported.^14^ The high-performance liquid chromatography (HPLC) chromatogram is shown in Figure S28, Supporting Information.

Compound purity was routinely checked by high-pressure liquid chromatography (HPLC). The purity of tested compounds was found to be >95%. The HPLC system used (Thermo Fisher Scientific Inc. Accela) coupled with a photodiode array (PDA) detector and an analytical automatic injection compartment. Samples were dissolved in acetonitrile (1 mg/mL). HPLC analysis was performed by using a Luna 5 μ C18 column (5 μm, 4.6 mm × 250 mm) at 25 ± 1 °C, with a solvent gradient elution (acetonitrile/water), a flow rate of 1.0 mL/min, and a signal detector at 254 nm. Eluent A (water/acetonitrile 95:5 + 0.05% formic acid), eluent B (acetonitrile/water 95:5 + 0.05% formic acid). Elution: 0–10 min up to 50% B; 5 min up to 100% B; 10 min at 100% B. HPLC chromatograms of compounds **5**–**10** and reference compound **3** (Figures S22–S28, Supporting Information) were acquired by the HPLC system consisting of a Thermo Scientific Accela chromatograph equipped with an automatic injector and a column heater coupled with a PDA detector. The analytical controls were performed on a Kinetex EVO 2.6 μm C18 100 Å (100 × 3,0 mm·ID) column (Phenomenex, Torrance, California) in gradient elution. Eluents: (A) H_2_O/ACN, 95/5 + 0,1% trifluoroacetic acid and (B) ACN + 0,1% trifluoroacetic acid. The gradient profile A (Table S2, Supporting Information) was used for compounds **3**, **5**, and **8** at a flow rate of 0.7 mL/min, room temp. The gradient profile B (Table S3, Supporting Information) was used for compounds **6**, **7**, **9**, and **10** at a flow rate of 0.7 mL/min, at room temperature. Samples were dissolved in the corresponding mobile phase, approximately at a concentration of 1 mg/mL. The injection volume was 2 μL.

#### Preparation of Compounds **5**–**10**

##### 4-Bromo-*N*-(4-(furan-2-yl)phenyl)benzenesulfonamide (**8**)

4-Bromobenzenesulfonyl chloride (**11**) (52 mg, 0.21 mmol) was dropped in an ice-cold solution of 4-(furan-2-yl)aniline (**15**) (30 mg, 0.19 mmol) in anhydrous pyridine (3 mL). The reaction was heated at 120 °C for 2 h while stirring under an argon stream. After cooling, the mixture was quenched with crushed ice, made acidic with 1 N hydrochloric acid, and extracted with ethyl acetate. The organic layer was washed with brine, dried over anhydrous sodium sulfate, and filtered. Evaporation of the solvent gave a residue that was purified by silica gel column chromatography (Hex/AcOEt, 6:4) to **8** (37 mg, 48%), mp 163–166 °C (from ethanol). ^1^H NMR (CDCl_3_): δ 6.38–6.39 (m, 1H), 6.46 (s, 1H), 6.52–6.53 (d, *J* = 3.3 Hz, 1H), 6.99–7.02 (m, 2H), 7.37–7.37 (m, 1H), 7.47–7.54 ppm (m, 6H). ^13^C NMR (DMSO-*d*_6_) δ 105.4, 112.0, 120.7, 124.4, 126.7, 126.9, 128.9, 136.5, 138.6, 142.8, 152.4 ppm. IR: ν 1137 and 2713 cm^–1^. The HPLC chromatogram is shown in Figure S25, Supporting Information.

##### 4-Bromo-*N*-(3-(furan-2-yl)phenyl)benzenesulfonamide (**5**)

4-Bromo-*N*-(3-(furan-2-yl)phenyl)benzenesulfonamide was synthesized as **8** starting from **11** and 3-(furan-2-yl)aniline (**13**). Yield 58%, mp 147–150 °C. ^1^H NMR (DMSO-*d*_6_): δ 6.57–6.58 (m, 1H), 6.86–6.87 (m, 1H), 6.98–7.01 (m, 1H), 7.26–7.30 (t, *J* = 7.9 Hz, 1H), 7.38–7.40 (m, 1H), 7.42–7.43 (t, *J* = 1.7, 1H), 7.67–7.71 (m, 2H), 7.74–7.74 (m, 1H), 7.76–7.79 (m, 2H) 10.48 ppm (s, 1H). ^13^C NMR (DMSO-*d*_6_) δ 106.9, 112.6, 115.3, 119.6, 120.1, 127.3, 129.1, 130.4, 131.7, 132.9, 138.5, 139.1, 143.7, 152.7 ppm. IR: ν 1191 and 2727 cm^–1^. The HPLC chromatogram is shown in Figure S22, Supporting Information

##### 4-Bromo-*N*-(3-(pyridin-2-yl)phenyl)benzenesulfonamide (**6**)

4-Bromo-*N*-(3-(pyridin-2-yl)phenyl)benzenesulfonamide was synthesized as **8** starting **11** from 3-(pyridine-2-yl)aniline (**14**). Yield 94%, mp 175–177 °C (from ethanol). ^1^H NMR (DMSO-*d*_6_): δ 7.15–7.17 (m, 1H), 7.34–7.38 (m, 2H), 7.68–7.78 (m, 5H), 7.83–7.87 (m, 3H), 8.64–8.65 (d, *J* = 4,8 Hz, 1H), 10.49 ppm (s, 1H). ^13^C NMR (DMSO-*d*_6_) δ 118.4, 120.2, 120.8, 122.6, 122,9, 126.6, 128,7, 129,7, 132.4, 137.4, 140.0, 138.7, 139.7, 149.6, 155.1 ppm. IR: ν 1156 and 2723 cm^–1^. The HPLC chromatogram is shown in Figure S23, Supporting Information.

##### 4-Chloro-*N*-(3-(pyridin-2-yl)phenyl)benzenesulfonamide (**7**)

4-Chloro-*N*-(3-(pyridin-2-yl)phenyl)benzenesulfonamide was synthesized as **8** starting from 4-chlorobenzenesulfonyl chloride (**12**) and **14**. Yield 28%, mp 166–169 °C (from ethanol). ^1^H NMR (CDCl_3_): δ 7.13–7.17 (m, 1H), 7.18–7.21 (m, 2H), 7.27–7.31 (m, 2H), 7.57, 7.60 (m, 3H), 7.63–7.71 (m, 3H), 8.58–8.60 ppm (m, 1H). ^13^C NMR (DMSO-*d*_6_) δ 118.4, 120.2, 120.8, 122.5, 122.9, 128.6, 129.5, 129.7, 137.4, 137.8, 138.0, 138.2, 139.7, 149.6, 155.1 ppm. IR: ν 1157 and 2790 cm^–1^. The HPLC chromatogram is shown in Figure S24, Supporting Information.

##### 4-Bromo-*N*-(4-(pyridin-2-yl)phenyl)benzenesulfonamide (**9**)

4-Bromo-*N*-(4-(pyridin-2-yl)phenyl)benzenesulfonamide was synthesized as **8** starting from **11** and 4-(pyridin-2-yl)aniline (**16**). Yield 56%, mp 162–165 °C (from ethanol). ^1^H NMR (CDCl_3_): δ 7.02 (s, 1H), 7.13–7.16 (d, *J* = 8.6 Hz, 2H), 7.21–7.24 (m, 1H), 7.54–7.57 (m, 2H), 7.62–7.66 (m, 3H), 7.72–7.76 (m, 1H), 7.85–7.88 (m, 2H), 8.66–8.68 ppm (m, 1H). ^13^C NMR (DMSO-*d*_6_) δ 119.8, 120,0, 127.0, 127.5, 128.7, 132.5, 134.5, 137.2, 138.3, 138.7, 149.5, 155.2 ppm. IR: ν 1155 and 2981 cm^–1^. The HPLC chromatogram is shown in Figure S26, Supporting Information.

##### 4-Chloro-*N*-(4-(pyridin-2-yl)phenyl)benzenesulfonamide (**10**)

4-Chloro-*N*-(4-(pyridin-2-yl)phenyl)benzenesulfonamide was synthesized as **8** starting from **12** and **16**. Yield 46%, mp 180–182 °C. ^1^H NMR (DMSO-d_6_): δ 7.10 (s, 1H), 7.13–7.16 (m, 2H), 7.21–7.24 (m, 1H), 7.37–7.41 (m, 2H), 7.63–7.66 (m, 1H), 7.69–7.76 (m, 3H), 7.85–7.87 (d, *J* = 8,6 Hz, 2H), 8.66–8.68 ppm (m, 1H). IR: ν 1162 and 2883 cm^–1^. The HPLC chromatogram is shown in Figure S27, Supporting Information.

#### General Procedure for the Preparation of Compounds **13**–**16**

##### Example: 4-(Furan-2-yl)aniline (**15**)

Tin(II) chloride diihydrate (662 mg, 2.93 mmol) was added to compound **19** (111 mg, 0.59 mmol) dissolved in ethyl acetate (5 mL). The reaction mixture was heated at 80 °C for 3 h. After cooling, the mixture was made basic with a saturated solution of sodium bicarbonate, and the resulting suspension was filtered. The organic layer was washed with brine, dried over anhydrous sodium sulfate, and filtered. Evaporation of the solvent gave a residue that was purified by silica gel column chromatography (CHex/AcOEt, 7:3) to give **15** (34 mg, 36%) as an oil. ^1^H NMR (CDCl_3_): δ 3.65 (br s, disappeared after treatment with D_2_O, 2H), 6.34–6.38 (m, 2H), 6.61–6.64 (m, 2H), 7.31–7.32 (m, 1H) and 7.39–7.42 ppm (m, 2H). IR: ν 1422 and 3426 cm^–1^.

##### 3-(Furan-2-yl)aniline (**13**)

3-(Furan-2-yl)aniline was synthesized as **15** starting from **17**. Yield 99% as and oil. ^1^H NMR (CDCl_3_): δ 3.63 (br s, disappeared after treatment with D_2_O, 2H), 6.37–6.38 (m, 1H), 6.50–6.53 (m, 2H), 6.94 (t, *J* = 2.0 Hz, 1H), 7.00 (dt, *J* = 1.4 and 7.8 Hz, 1H), 7.09 (t, *J* = 7.8 Hz, 1H) and 7.36–7.37 ppm (m, 1H). IR: ν 1456 and 3424 cm^–1^.

##### 3-(Pyridin-2-yl)aniline (**14**)

3-(Pyridin-2-yl)aniline was synthesized as **15** starting from **18**. Yield 50%, mp 64–66 °C. ^1^H NMR (DMSO-*d*_6_): δ 5.18 (br s, disappeared after treatment with D_2_O, 2H), 6.62–6.64 (m, 1H), 7.12 (t, *J* = 7.7 Hz, 1H), 7.17–7.20 (m, 1H), 7.29–7.32 (m, 1H), 7.34–7.35 (m, 1H), 7.79–7.86 (m, 2H) and 8.61–8.63 ppm (1H). IR v 1457 and 3418 cm^–1^.

##### 4-(Pyridin-2-yl)aniline (**16**)

4-(Pyridin-2-yl)aniline was synthesized as **15** starting from **20**. Yield 65%, mp 93–96 °C (mp 89–92 °C), Lit.^46^^1^H NMR (DMSO-*d*_6_): δ 5.41 (br s, disappeared after treatment with D_2_O, 2H), 6.63 (d, *J* = 8.6 Hz, 2H), 7.14–7.18 (m, 1H), 7.71–7.76 (m, 2H), 7.80 (d, *J* = 8.6 Hz, 2H) and 8.51–8.53 ppm (m, 1H). IR: 1469 and 3185 cm^–1^.

#### Preparation of Compounds **17**–**20**

##### 2-(4-Nitrophenyl)furan (**19**)

To a mixture of 1-bromo-4-nitrobenzene (**22**) (500 mg, 2.48 mmol) and furan-2-boronic acid (**23**) (388 mg, 3.47 mmol) in tetrahydrofuran (30 mL) was added a solution of sodium carbonate (0.67 g, 6.19 mmol) in water (10 mL). The mixture was degassed for 15 min, and then [1,1]-[bis(diphenylphosphino)ferrocene]dichloropalladium(II) (127 mg, 0.17 mmol) was added. The reaction mixture was heated at reflux for 1.5 h under an argon stream. After cooling, the mixture was diluted with water and extracted with AcOEt. The organic layer was washed with brine, dried over anhydrous sodium sulfate, and filtered. Evaporation of the solvent gave a residue that was purified by silica gel column chromatography (cHex/AcOEt, 7:3) to give **19** (139 mg, 30%) as an oil. ^1^H NMR (DMSO-*d*_6_): δ 6.72–6.73 (m, 1H), 7.33 (dd, *J* = 0.8 and 3.6 Hz, 1H), 7.94 (dd, *J* = 0.8 and 1.8 Hz, 1H), 7.95–7.98 (m, 2H) and 8.27–8.31 ppm (2H). IR: ν 1389 and 2986 cm^–1^.

##### 2-(3-Nitrophenyl)furan (**17**)

2-(3-Nitrophenyl)furan was synthesized as **19** starting from 1-bromo-3-nitrobenzene (**21**) and **23**. Yield 80% as an oil. ^1^H NMR (DMSO-*d*_6_): δ 6.67–6.69 (m, 1H), 7.26–7.27 (m, 1H), 7.72 (t, *J* = 8.0 Hz, 1H), 7.86–7.87 (m, 1H), 8.11–8.16 (m, 2H) and 8.45 ppm (t, *J* = 2.0 Hz, 1H). IR: ν 1377 and 3022 cm^–1^.

##### 2-(3-Nitrophenyl)pyridine (**18**)

2-(3-Nitrophenyl)pyridine was synthesized as **19** starting from 2-iodopyridine (**24**) and (3-nitrophenyl)boronic acid (**25**). Yield 67%, mp 61–64 °C. ^1^H NMR (DMSO-*d*_6_): δ 7.45–7.49 (m, 1H), 7.81 (t, *J* = 8.0 Hz, 1H), 7.98 (td, *J* = 1.8 and 7.7 Hz, 1H), 8.16 (dt, *J* = 0.8 and 8.0 Hz, 1H), 8.28–8.31 (m, 1H), 8.54–8.56 (m, 1H), 8.74–8.76 (m, 1H) and 8.92–8.93 ppm (m, 1H). IR: ν 1346 and 3090 cm^–1^.

##### 2-(4-Nitrophenyl)pyridine (**20**)

2-(4-Nitrophenyl)pyridine was synthesized as **19** starting from **24** and (4-nitrophenyl)boronic acid (**26**). Yield 57%, mp 128–131 °C (mp 131–133 °C, Lit.^46^). ^1^H NMR (DMSO-d_6_): δ 7.47–7.51 (m, 1H), 7.99 (td, *J* = 1.9 and 7.7 Hz, 1H), 8.15 (dt, *J* = 1.0 and 8.0 Hz, 1H), 8.33–8.40 (m, 4H) and 8.75–8.77 ppm (m, 1H). IR: ν 1343 and 2981 cm^–1^.

### Crystallography

#### Protein Expression and Purification

The β-catenin Armadillo repeats domain (aa 133–664) was expressed as the N-terminally GST-tagged protein using the pGEX-4T-1 vector containing a TEV cleavage site. The expression plasmid was used to transform *Escherichia coli* BL21(DE3) cells; the culture was grown at 37 °C in LB medium with 100 μg/mL Ampicillin until OD_600_ = 0.6–0.7, and then protein expression was induced with 0.5 mM IPTG (Merck) and the cells were cultured overnight at 18 °C. The cell pellet was collected by centrifugation and resuspended in PBS supplemented with a protease inhibitor cocktail (Roche). Prior to sonication, Triton X-100 1% (v/v) was added, gently shaken, and incubated for 30′ at 22 °C for complete protein solubilization. The supernatant was then loaded onto a glutathione agarose resin (Thermo Scientific) and the eluted GST-fusion protein was incubated overnight at 20 °C with TEV protease (ratio 1:30) for tag removal. The protein was further purified by monoQ ion exchange (Sigma-Aldrich) in 20 mM Tris–HCl buffer, pH 8.8, 2 mM DTT, 2% (v/v) glycerol with a 0–1.0 M NaCl linear gradient. The pooled fraction containing the pure protein was then concentrated for crystallization at 4.5 mg/mL using Amicon centrifugal concentrators (Millipore) with a 10 kDa cutoff membrane.

#### Crystallization and Data Collection

Needle-shaped crystals were obtained by vapor diffusion at 20 °C using a hanging drop made by mixing 2 μL of protein solution with 2 μL of reservoir solution (7–9% (w/v) PEG 6K, 100 mM Na Citrate, pH 5.6, 5 mM DTT, 15% (v/v) isopropanol). The crystals were soaked for a few days in a storage solution (10% (w/v) PEG 6K, 100 mM Na citrate, pH 5.6, 5 mM DTT, 15% (v/v) isopropanol) containing ligand **9** at a concentration of 1 mM. The ligand, dissolved in DMSO, was diluted in the storage solution so that the final concentration of DMSO was 2%. According to the literature, a solution of 15 mM Tris–HCl, pH 8.5, 80 mM NaCl, 80 mM Na citrate, pH 5.6, 15% (v/v) isopropanol, 22% (w/v) PEG 6K, AND 15% (v/v) ethylene glycol was used as cryoprotectant. Crystals of Armadillo/**9** belong to space group P2(1) with cell parameters shown in Table S1, Supporting Information.

#### Structure Determination and Refinement

The X-ray data set was collected at 100 K under a nitrogen stream using synchrotron radiation of the Elettra XRD2 beamline (Trieste, Italy). The collected data were processed using X-ray detector software (XDS) and Scala.^47^ The structure solution was prepared with AmoRe,^48^ using the coordinates of β-catenin in a complex with a phosphorylated APC 20aa repeats fragment^49^ (PDB ID 1TH1) as the starting model. The coordinates were then refined with CNS^50^ and Phenix,^51^ including data between 50 and 3.43 Å. The statistics of crystallographic data and refinement are summarized in Table 1. The coordinates and structure factors of the complex have been deposited in the PDB under accession number 7ZRB.

#### Surface Plasmon Resonance

SPR experiments were performed at 25 °C using a Pioneer AE optical biosensor (Sartorius) equipped with a PCH sensor chip (linear polycarboxylate hydrogel layer) and equilibrated with running buffer 10 mM Hepes, pH 7.4, 150 mM NaCl, 0.005% Tween-20, and 1% DMSO. The PCH sensor chip was installed and conditioned in accordance with the manufacturer’s instructions.

The β-catenin Armadillo domain was immobilized on the surface using amine coupling chemistry.^52^ Briefly, flow cells were activated for 4 min by injecting 40 μL of a 1:1 ratio of 100 mM *N*-hydroxysuccinimide (NHS)/400 mM ethyl-3(3-dymethylamino) propyl carbodiimide (EDC). The protein solution (100 μg mL^–1^ in 10 mM Na Acetate, pH 4.5) was then injected at 10 μL/min, followed by a 70 μL injection of ethanolamine 1 M, pH 8.0, to block any remaining activated groups on the surface. Approx. 11,100 and 9000 RUs of protein were immobilized on CH1 and CH3 of the sensor chip, respectively, whereas CH2 was used as a reference for nonspecific binding.

The stability of the surface was demonstrated by the flat baseline achieved at the beginning (0–60 s) of each sensorgram. The analyte **9** was dialyzed in the running buffer (1% final DMSO concentration) and injected at different concentrations for 120 s onto the sensor chip at a constant flow rate of 50 μL/min. For each analyte injection, a dissociation of 180 s was allowed, followed by a short mild regeneration step with 10 mM NaOH and 1 M NaCl. All sensorgrams were processed using double referencing. First, responses from the reference surface (CH2) were subtracted from the binding responses collected over the reaction surfaces to correct for bulk refractive index changes between the flow buffer and analyte sample. Second, the response from the closest blank injection (zero analyte concentration) was subtracted to compensate for drift and small differences between the activated channel and the reference flow cell CH2. To obtain kinetic rate constants and affinity constants, the corrected response data were fitted in QDAT software provided with the instrument (Biologic Software). A kinetic analysis of the ligand/analyte interaction was obtained by fitting the response data to a reversible 1:1 bimolecular interaction model. The equilibrium dissociation constant (*K*_D_) was determined by the ratio *K*_off_/*K*_on_. This experiment was performed in duplicate.

### Molecular Modeling

All molecular modeling studies were performed on a MacPro dual 2.66 GHz Xeon running Ubuntu 20 LTS. The protein was prepared by Maestro protein preparation wizard Sastry.^53^ Ligand structures were built with Maestro and minimized using the MMFF94× force field. The docking computations were performed by Plants. The water molecules highlighted to be crucial for the binding were included in the docking studies. The images in the manuscript were created with PyMOL.^54^

### Biology

#### Cell Cultures

SW480 and HCT116 cells were grown in DMEM (41965-039, GIBCO, Thermo Fisher Scientific, Waltham, MA) supplemented with 10% FBS (10270, GIBCO, Thermo Fisher Scientific, Waltham, MA), glutamax (35050-061, GIBCO, Thermo Fisher Scientific, Waltham, MA), and pen/strep (15070-063, GIBCO, Thermo Fisher Scientific, Waltham, MA). HEK293 transfection was performed using lipofectamine 2000 (11668019, Thermo Fisher Scientific, Waltham, MA) following the manufacturer’s instructions. Mother stock solutions of compounds were obtained by dissolving them in DMSO at a dilution of 30 mM and storing them at −80 °C. MDA-MB-231, HCC1806, and HCC1937 TNBC cells were purchased from ATCC and grown in RPMI 1640 (Lonza), supplemented with 10% FBS, gentamycin, penicillin, and streptomycin.

#### Cell Viability

Cell viability was evaluated by XTT^55^ (SW480 cells) or MTT^56^ (HCT116 cells) colorimetric assays. Briefly, cells (range 10–30 × 10^3^ cells/well) were seeded in 96-well microculture plates and then exposed to increasing concentrations of different compounds (range 0–300 μM) for 48 or 72 h. At the end of the treatment, media were removed and incubated at 37 °C in the dark for 4 h in phosphate-buffered saline (PBS) containing 0.2 mg/mL sodium 3′-[1-[(phenylamino)-carbonyl]-3,4-tetrazolium]-bis(4-methoxy-6-nitro)benzene-sulfonic acid hydrate (XTT) (Thermo Fisher Scientific, Waltham, MA) or 3-[4,5-dimethylthiazol-2-yl]-2,5 diphenyl tetrazolium bromide (MTT) (Merck, Kenilworth, NJ) and phenazine methosulfate (PMS) at a final concentration of 25 μM. Absorbance at 450 nm and a reference wavelength of 650 nm was then measured using a microplate spectrophotometer (Multiskan FC Microplate Photometer, Thermo Scientific, Waltham, MA). The cell growth inhibition rate was calculated utilizing the following formula: inhibition rate (%) = [Control OD – (Sample OD/Control OD)] × 100, where Control OD is the absorbance of the negative control and Sample OD is the absorbance of the test sample. The IC_50_ values were determined with GraphPad Prism 5 through constructed dose–response curves.

Cell proliferation of breast cancer cell lines upon continuous treatment with select compounds was evaluated by the real-time quantitative live-cell imaging system. MDA-MB-231 (1500 cells/well), HCC1806 (2000 cells/well), and HCC1937 (3000 cells/well) were seeded in 96-well plates, treated with increasing doses of selected compounds and images (5 images/well) were acquired every 8 h for 3 days at 10× magnification in an IncuCyte SX5 live-content imaging system (Essen Bioscience) at 37 °C with 5% CO_2_. Images were analyzed using IncuCyte cell-by-cell analysis software to detect and quantify live cells (phase-contrast). The number of cells at each time point was quantified using IncuCyte cell-by-cell analysis software, and cell growth was evaluated as the normalized ratio between counted cells at each time point normalized to those counted at time 0. Graph drawing and statistical analyses were performed in GraphPad Prism according to the statistical tests described in the figure legends.

#### Topflash Dependent Luciferase Activity

According to the report,^57^ the luciferase report assay of Topflash/Foplash was used to measure the activity of the Wnt/β-catenin signaling pathway. Briefly, the Topflash plasmid (Beyotime, Zhejiang, China) contains 7 TCF/LEF binding sites, while the Fopflash plasmid (Beyotime, Zhejiang, China) contains 6 mutated TCF/LEF binding sites, which are located in the upstream of the luciferase reporter. SW480 and HCT116 cell lines were cultured in 90% DMEM (HyClone, #SH30243.01) plus 10% fetal bovine serum (Gibco, #10270) and plasmocin (mpp-39-03, InvivoGen), respectively. The Topflash/Fopflash vector and plasmid pTK-renilla together were co-transfected to the cells. The cells were treated with each β-catenin inhibitor (IC_50_ concentration) for 12 h. The Dual-Glo luciferase assay kit (Promega, #E1910) was used to measure the luminescence intensity (Figure 2).

#### Wnt Reporter Assay

Luciferase assays were performed as previously described.^58,59^ Briefly, HCT116 cells were transfected with Topflash reporter (M50 Super 8x TOPFlash, containing 8 repeats of TCF/LEF binding sites) or Fopflash reporter (M51 Super 8x FOPFlash, containing the mutated TCF/LEF binding sites) plasmids using the Dreamfect Gold Transfection Reagent (#DG80500, OZ Biosciences). After overnight incubation, cells were starved for 8 h in a medium containing Opti-MEM (#31985070, Thermo Fisher Scientific), FBS 0.5%, pen/strep 1%, NaPyr 1%, and NeAA 1% for 8 h and then co-treated with 50 mM LiCl or DMSO and the indicated amounts of compound 4 or 14 for 24 h. The luciferase assay was performed at the end of the treatment, and dose–response curves were generated using Graphpad Prism software.

#### Co-Immunoprecipitation Assay

Co-immunoprecipitation assays were performed as previously described.^16,60^ Briefly, 2 × 10^4^/cm^2^ HCT116 cells were seeded in a 100 mm dish and incubated overnight at 37 °C. Cells were transfected with pcDNA/Myc Tcf-4 (Addgene plasmid #16512; http://n2t.net/addgene:16512; RRID:Addgene_16512)^29^ and, after overnight incubation, were starved for 8 h in a medium containing Opti-MEM (#31985070, Thermo Fisher Scientific), FBS 0.5%, pen/strep 1%, NaPyr 1%, NeAA 1%, and then co-treated with 50 mM LiCl and DMSO or 50 μM compound **9** or 50 μM compound 6454 for 24 h. Cells were collected at the end of the treatments and lysed in a buffer containing 0.5% Triton X-100, 0.5 mM EDTA, and 1 mM DTT. Protein extracts were incubated with 20 μL of immobilized anti-c-myc beads (#sc-40 AC, Santa Cruz Biotechnology) and mixed overnight at 4 °C. Complexes were washed and the c-Myc-tagged Tcf-4 protein was eluted with reducing sample buffer. Western blot analysis was performed using an anti-c-Myc antibody (#M4439, Sigma-Aldrich) to detect Myc-tagged Tcf-4 and an anti-β-catenin antibody to reveal endogeneous β-catenin (#sc-7963, Santa Cruz Biotechnology). Chemioluminescence was detected using WesternBright ECL (#K-12045-D50, Advansta), according to the manufacturer’s protocol.

#### In Vivo Xenograft Experiments

Briefly, all 10-week-old female BALB/C^nu/nu^ mice (8 mice) were purchased from the Shanghai University of Traditional Chinese Medicine with Institutional Animal Care and Use Committee approval in accordance with institutional guidelines. All mice were randomly divided into four groups. In the #1 group (4 mice), 1 × 10^8^ cells/mL from HCT116 at the logarithmic growth phase were harvested and inoculated subcutaneously into BALB/C^nu/nu^ mice and intraperitoneally injected with 100 μL of compound **9** (25 mg/kg) every 2 days after tumorigenesis. In the #2 group (4 mice), 1 × 10^8^ cells/mL from compound **9** at the logarithmic growth phase were harvested and inoculated subcutaneously into BALB/C^nu/nu^ mice and intraperitoneally injected 100 μL of saline every 2 days after tumorigenesis. After continuous feeding for 30 days, the mice were sacrificed, and the tumors were removed. The tumors were weighed, and the volumes were calculated using the following formula: tumor volume (cm^3^) = (*ab*^2^)/2 (*a*: the longest axis (cm), *b*: the shortest axis (cm))

#### Hematoxylin and Eosin Staining

Tissue samples were fixed in 4% paraformaldehyde, dehydrated, and embedded in paraffin. The paraffin-embedded tissues were cut into 4 μm sections using a microtome, and the sections were affixed onto glass slides. Subsequently, the sections were dewaxed using xylene and subjected to dehydration in an ethanol gradient. The sections were stained with hematoxylin (H) for 5 min at room temperature, and then 1% ethanol was added for 30 s for differentiation. Afterward, aqueous ammonia was added for 1 min for blueing, followed by rinsing in distilled water for 5 min. Subsequently, the sections were stained with eosin (E) for 2 min at room temperature and then rinsed with distilled water for 2 min. Then, decolorization over an ethanol gradient was performed, and xylene was added for 2 min for clearing. Finally, the sections were sealed and mounted with neutral resin.

#### Immunofluorescence Staining

Briefly, fresh tissues were immersed in 4% paraformaldehyde (Sigma-Aldrich) for fixation at room temperature for 30 min. The tissues were then dehydrated in an ethanol gradient, embedded in paraffin, sectioned (thickness: 6 μm), and immersed in xylene for dewaxing. Tissue sections were blocked with an immunohistochemical blocking solution (Beyotime Biotechnology Co., Ltd., Zhejiang, China) at 37 °C for 30 min. The blocking solution was then discarded, and the sections were washed 3 times at room temperature for 5 min each with an immunohistochemical washing solution (Beyotime Biotechnology). Then, primary antibodies (rabbit anti-Ki67 antibody (ab15580), Abcam, MA) were added and incubated at 37 °C for 45 min. After incubation, the antibody solution was discarded, and the sections were washed 3 times at room temperature for 5 min each with the immunohistochemical washing solution (Beyotime Biotechnology). Then, secondary antibodies (goat anti-rabbit IgG H&L (Alexa Fluor 488), Abcam, MA) were added and the tissues were incubated at 37 °C for 45 min. After incubation, the antibody solution was discarded, and the sections were washed 3 times at room temperature for 5 min each with the immunohistochemical washing solution (Beyotime Biotechnology). Finally, an immunofluorescence blocking solution (Sigma-Aldrich) was added, and the sections were mounted.

#### Western Blot

Briefly, total proteins from the cells in each group were subjected to 12% denaturing sodium dodecyl sulfate-polyacrylamide gel electrophoresis (SDS-PAGE). The proteins were then transferred onto a poly(vinylidene fluoride) (PVDF) membrane (Millipore). The membrane was blocked, washed, and incubated with primary antibodies at 37 °C for 45 min (rabbit anti-human β-catenin (D10A8) XP mAb (#8480), rabbit anti-human phospho-β-catenin (Ser675) (D2F1) XP mAb (#4176), rabbit anti-human Wnt3a (C64F2) mAb (#2721), rabbit anti-human Wnt5a/b (C27E8) mAb (#2530), Cell Signaling Technology, MA; rabbit anti-GAPDH antibody [EPR16891] (ab181602), Abcam, MA). After the membrane was fully washed, it was incubated with secondary antibodies at 37 °C for 45 min goat anti-rabbit IgG H&L (HRP) (ab97051), Abcam, MA. The membrane was washed with Tris-buffered saline/Tween-20 (TBST) 4 times at room temperature for 14 min each time. Next, the samples were exposed and imaged (Sigma-Aldrich Chemical) using the enhanced chemiluminescence (ECL) method (Pierce Biotechnology).

## Supporting Information Available

2Fo-Fc electron density map of compound **9** countered at 0.8 σ (Figure S1); docking proposed binding modes of derivative **9** and ref (3) (Figure S2); inhibition of MDA-MB-231 cells (Figures S3–S11); inhibition of HCC1806-231 cells (Figures S12–S19); inhibition of HCC1937 cells (Figures S20 and S21); HPLC chromatogram of compound **9** (Figure S22); HPLC chromatograms of compounds **3** and **5**–**10** (Figures S21–S28); statistics of crystallographic data (Table S1); and HPLC gradient profiles A and B (Tables S2 and S3) (PDF)

## Abbreviations

APC: adenomatous polyposis coli
CRC: colorectal cancer
DMSO: dimethyl sulfoxyde
LEF: lymphoid enhancer-binding factor
NHERF1: Na^+^/H^+^ exchanger 3 regulating factor 1
TCF: T-cell factor
TNBC: triple-negative breast cancer
Wnt: wingless/integrase-1
β-TrCP: β-transducing repeats-containing proteins

## References

[ref1] Clevers H.; Nusse R. Wnt/β-catenin signaling and disease. Cell 2012, 149, 1192–1205. 10.1016/j.cell.2012.05.012. 22682243

[ref2] Li V. S.; Ng S. S.; Boersema P. J.; Low T. Y.; Karthaus W. R.; Gerlach J. P.; Mohammed S.; Heck A. J.; Maurice M. M.; Mahmoudi T.; Clevers H. Wnt signaling inhibits pro-teasomal β-catenin degradation within a compositionally intact Axin1 complex. Cell 2012, 149, 1245–1256. 10.1016/j.cell.2012.05.002. 22682247

[ref3] Clevers H.; Loh K. M.; Nusse R. Stem cell signaling. An integral program for tissue renewal and regeneration: Wnt signaling and stem cell control. Science 2014, 346, 1248012 10.1126/science.1248012. 25278615

[ref4] Zhang Y.; Wang X. Targeting the Wnt/β-catenin signaling pathway in cancer. J. Hematol. Oncol. 2020, 13, 165 10.1186/s13045-020-00990-3. 33276800 PMC7716495

[ref5] Nagase H.; Nakamura Y. Mutations of the APC (adenomatous polyposis coli) gene. Hum. Mutat. 1993, 2, 425–434. 10.1002/humu.1380020602. 8111410

[ref6] Zhang H.; Liu C.; Zhu D.; Zhang Q.; Li J. Medicinal chemistry strategies for the development of inhibitors disrupting β-catenin’s interactions with its nuclear partners. J. Med. Chem. 2023, 66, 1–31. 10.1021/acs.jmedchem.2c01016. 36583662

[ref7] Shang S.; Hua F.; Hu Z.-W. The regulation of β-catenin activity and function in cancer: therapeutic opportunities. Oncotarget 2017, 8, 33972–33989. 10.18632/oncotarget.15687. 28430641 PMC5464927

[ref8] Yan M.; Li G.; An J. Discovery of small molecule inhibitors of the Wnt/β-catenin signaling pathway by targeting β-catenin/Tcf4 interactions. Exp. Biol. Med. 2017, 242, 1185–1197. 10.1177/1535370217708198. PMC5478005 28474989

[ref9] Siegel R. L.; Miller K. D.; Fuchs H. E.; Jemal A. Cancer statistics, 2022. Ca-Cancer J. Clin. 2022, 72, 7–33. 10.3322/caac.21708. 35020204

[ref10] Cassidy S.; Syed B. A. Colorectal cancer drugs market. Nat. Rev. Drug Discovery 2017, 16, 525–526. 10.1038/nrd.2017.59. 28529321

[ref11] Xie Y.-H.; Chen Y.-X.; Fang J.-Y. Comprehensive review of targeted therapy for colorectal cancer. Signal Transduction Targeted Ther. 2020, 5, 22 10.1038/s41392-020-0116-z. PMC7082344 32296018

[ref12] Nalli M.; Puxeddu M.; La Regina G.; Gianni S.; Silvestri R. Emerging therapeutic agents for colorectal cancer. Molecules 2021, 26, 7463 10.3390/molecules26247463. 34946546 PMC8707340

[ref13] Saponaro C.; Sergio S.; Coluccia A.; De Luca M.; Mologni L.; Vergar D.; Salzet M.; Fournier I.; Bucci C.; Bonetti D.; Gautier C.; Gianni S.; Silvestri R.; Famiglini V.; Naccarato V.; Passerini C. G.; Maffia M.; Coluccia A. M. L. β-Catenin knockdown promotes NHERF1-mediated survival of colorectal cancer cells: implications for a double-targeted therapy. Oncogene 2018, 37, 3301–3316. 10.1038/s41388-018-0170-y. 29551770 PMC6002344

[ref14] Di Magno L.; Di Pastena F.; Puxeddu M.; La Regina G.; Coluccia A.; Ciogli A.; Manetto S.; Maroder M.; Canettieri G.; Silvestri R.; Nalli M. Sulfonamide inhibitors of β-catenin signaling with different output on c-MYC as anticancer agents. ChemMedChem 2020, 15, 2264–2268. 10.1002/cmdc.202000594. 32946182

[ref15] Handeli S.; Simon J. A. A small-molecule inhibitor of Tcf/beta-catenin signaling down regulates PPARgamma and PPARdelta activities. Mol. Cancer Ther. 2008, 7, 521–529. 10.1158/1535-7163.MCT-07-2063. 18347139

[ref16] Coluccia A.; La Regina G.; Naccarato V.; Nalli M.; Orlando V.; Biagioni S.; De Angelis M. L.; Baiocchi M.; Gautier C.; Gianni S.; Di Pastena F.; Di Magno L.; Canettieri G.; Coluccia A. M. L.; Silvestri R. Drug design and synthesis of first in class PDZ1 targeting NHERF1 inhibitors as anticancer agents. ACS Med. Chem. Lett. 2019, 10, 499–503. 10.1021/acsmedchemlett.8b00532. 30996786 PMC6466550

[ref17] Hwang S. Y.; Deng X.; Byun S.; Lee C.; Lee S. J.; Suh H.; Zhang J.; Kang Q.; Zhang T.; Westover K. D.; Mandinova A.; Lee S. W. Direct targeting of β-catenin by a small molecule stimulates proteasomal degradation and suppresses oncogenic Wnt/β-catenin signaling. Cell Rep. 2016, 16, 28–36. 10.1016/j.celrep.2016.05.071. 27320923 PMC4957947

[ref18] Brown N. Bioisostres in Medicinal Chemistry; Wiley-Vch Verlag: Weinheim, Germany, 2012.

[ref19] Ciapetti P.; Giethlen B. Molecular Variations Based on Isosteric Replacement. Carboxylic Esters Bioisosteres. In The Practice of Medicinal Chemistry, 3rd ed.; Wermuth C. G., Ed.; Elsevier Ltd: Oxford, U.K, 2008; pp 310–313 9780080568775.

[ref20] La Regina G.; Bai R.; Rensen W.; Coluccia A.; Piscitelli F.; Gatti V.; Bolognesi A.; Lavecchia A.; Granata I.; Porta A.; Maresca B.; Soriani A.; Iannitto M. L.; Mariani M.; Santoni A.; Brancale A.; Ferlini C.; Dondio G.; Varasi M.; Mercurio C.; Hamel E.; Lavia P.; Novellino E.; Silvestri R. Design and synthesis of 2-heterocyclyl-3- arylthio-1H-indoles as potent tubulin polymerization and cell growth inhibitors with improved metabolic stability. J. Med. Chem. 2011, 54, 8394–8406. 10.1021/jm2012886. 22044164 PMC3261769

[ref21] McCoy M. A.; Spicer D.; Wells N.; Hoogewijs K.; Fiedler M.; Baud M. G. J. Biophysical survey of small-molecule β-catenin inhibitors: a cautionary tale. J. Med. Chem. 2022, 65, 7246–7261. 10.1021/acs.jmedchem.2c00228. 35581674 PMC9150122

[ref22] Kessler D.; Mayer M.; Zahn S. K.; Zeeb M.; Wöhrle S.; Bergner A.; Bruchhaus J.; Ciftci T.; Dahmann G.; Dettling M.; Döbel S.; Fuchs J. E.; Geist L.; Hela W.; Kofink C.; Kousek R.; Moser F.; Puchner T.; Rumpel K.; Scharnweber M.; Werni P.; Wolkerstorfer B.; Breitsprecher D.; Baaske P.; Pearson M.; McConnell D. B.; Böttcher J. Getting a grip on the undrugged: Targeting β-catenin with fragment-based methods. ChemMedChem 2021, 16, 1420–1424. 10.1002/cmdc.202000839. 33275320 PMC8247886

[ref23] Tomasello G.; Armenia I.; Molla G. The Protein Imager: a full-featured online molecular viewer interface with server-side HQ-rendering capabilities. Bioinformatics 2020, 36, 2909–2911. 10.1093/bioinformatics/btaa009. 31930403

[ref24] Yu B.; Huang Z.; Zhang M.; Darren R. D. R.; Ji H. Rational design of small-molecule inhibitors for β-catenin/T-cell factor protein-protein interactions by bioisostere replacement. ACS Chem. Biol. 2013, 8, 524–529. 10.1021/cb300564v. 23272635

[ref25] Fasolini M.; Wu X.; Flocco M.; Trosset J. Y.; Oppermann U.; Knapp S. Hot spots in Tcf4 for the interaction with beta-catenin. J. Biol. Chem. 2003, 278, 21092–21098. 10.1074/jbc.M301781200. 12657632

[ref26] Cui C.; Zhou X.; Zhang W.; Qu Y.; Ke X. Is β-catenin a druggable target for cancer therapy?. Trends Biochem. Sci. 2018, 43, 623–634. 10.1016/j.tibs.2018.06.003. 30056837

[ref27] Coluccia A.; Bufano M.; La Regina G.; Puxeddu M.; Toto A.; Paone A.; Bouzidi A.; Musto G.; Badolati N.; Orlando V.; Biagioni S.; Masci D.; Cirilli R.; Cantatore C.; Cutruzzolà F.; Gianni S.; Stornaiuolo M.; Silvestri R. *S)*-5-Chloro-3-((3,5-dimethylphenyl)sulfonyl)-N-(1-oxo-1-((pyridin-4-ylmethyl)amino)propan-2-yl),1H-indole-2-carboxamide (RS4690) as a new Dishevelled 1 inhibitor. Cancers 2022, 14, 1358 10.3390/cancers14051358. 35267666 PMC8909805

[ref28] Tanaka H.; Kawaguchi M.; Shoda S.; Miyoshi T.; Iwasaki R.; Hyodo F.; Mori T.; Hara A.; Tomita H.; Matsuo M. Radioresistance and stemness in human colon cancer. Anticancer Res. 2019, 39, 6575–6583. 10.21873/anticanres.13873. 31810923

[ref29] Korinek V.; Barker N.; Morin P.; van Wichen D.; de Weger R.; Kinzler K.; Vogelstein B.; Clevers H. Constitutive transcriptional activation by a β-catenin-Tcf complex in APC-/- colon carcinoma. Science 1997, 275, 1784–1787. 10.1126/science.275.5307.1784. 9065401

[ref30] Morin P.; Sparks A.; Korinek V.; Barker N.; Clevers H.; Vogelstein B.; Kinzler K. Activation of β-catenin-Tcf signaling in colon cancer by mutations in β-catenin or APC. Science 1997, 275, 1787–1790. 10.1126/science.275.5307.1787. 9065402

[ref31] Arnold A.; Tronser M.; Sers C.; Ahadova A.; Endris V.; Mamlouk S.; Horst D.; Möbs M.; Bischoff P.; Kloor M.; Bläker H. The majority of β-catenin mutations in colorectal cancer is homozygous. BMC Cancer 2020, 20, 1038 10.1186/s12885-020-07537-2. 33115416 PMC7594410

[ref32] Mhaidat N. M.; Bouklihacene M.; Thorne R. F. 5-Fluorouracil-induced apoptosis in colorectal cancer cells is caspase-9-dependent and mediated by activation of protein kinase C-δ. Oncol. Lett. 2014, 8, 699–704. 10.3892/ol.2014.2211. 25013487 PMC4081407

[ref33] Reyland M. E.; Anderson S. M.; Matassa A. A.; Barzen K. A.; Quissell D. O. Protein kinase C delta is essential for etoposide-induced apoptosis in salivary gland acinar cells. J. Biol. Chem. 1999, 274, 19115–19123. 10.1074/jbc.274.27.19115. 10383415

[ref34] Brenton J. D.; Carey L. A.; Ahmed A. A.; Caldas A. Molecular classification and molecular forecasting of breast cancer: ready for clinical application?. J. Clin. Oncol. 2005, 23, 7350–7360. 10.1200/JCO.2005.03.3845. 16145060

[ref35] Anders C. K.; Carey L. A. Biology, metastatic patterns, and treatment of patients with triple-negative breast cancer. Clin. Breast Cancer 2009, 9, S73–S81. 10.3816/CBC.2009.s.008. 19596646 PMC2919761

[ref36] On-Yu H.; Eun-Mi N.; Hye-Yeon J.; Young-Rae L.; Byoung K. L.; Sung H. J.; Jong-Suk K.; Hyun J. Y. Epigallocatechin gallate inhibits the growth of MDA-MB-231 breast cancer cells via inactivation of the β-catenin signaling pathway. Oncol. Lett. 2017, 14, 441–446. 10.3892/ol.2017.6108. 28693189 PMC5494649

[ref37] Garrido-Castro A. C.; Lin N. U.; Polyak K. Insights into molecular classifications of triple-negative breast cancer: improving patient selection for treatment. Cancer Discovery 2019, 9, 176–198. 10.1158/2159-8290.CD-18-1177. 30679171 PMC6387871

[ref38] Li L. T.; Jiang G.; Chen Q.; Zheng J. N. Ki67 is a promising molecular target in the diagnosis of cancer (Review). Mol. Med. Rep. 2015, 11, 1566–1572. 10.3892/mmr.2014.2914. 25384676

[ref39] Schlüter A.; Weller P.; Kanaan O.; Nel I.; Heusgen L.; Höing B.; Haßkamp P.; Zander S.; Mandapathil M.; Dominas N.; Arnolds J.; Stuck B. A.; Lang S.; Bankfalvi A.; Brandau S. CD31 and VEGF are prognostic biomarkers in early-stage, but not in late-stage, laryngeal squamous cell carcinoma. BMC Cancer 2018, 18, 272 10.1186/s12885-018-4180-5. 29523110 PMC5845191

[ref40] De Jong J. S.; van Diest P. J.; Baak J. P. Heterogeneity and reproducibility of microvessel counts in breast cancer. Lab. Invest. 1995, 73, 922–926. 8558855

[ref41] Daina A.; Michielin O.; Zoete V. SwissADME: a free web tool to evaluate pharmacokinetics, drug-likeness and medicinal chemistry friendliness of small molecules. Sci. Rep. 2017, 7, 42717 10.1038/srep42717. 28256516 PMC5335600

[ref42] Lipinski C. A.; Lombardo F.; Dominy B. W.; Feeney P. J. Experimental and computational approaches to estimate solubility and permeability in drug discovery and development settings. Adv. Drug Delivery Rev. 1997, 23, 3–26. 10.1016/S0169-409X(96)00423-1. 11259830

[ref43] Veber D. F.; Johnson S. R.; Cheng H. Y.; Smith B. R.; Ward K. W.; Kopple K. D. Molecular properties that influence the oral bioavailability of drug candidates. J. Med. Chem. 2002, 45, 2615–2623. 10.1021/jm020017n. 12036371

[ref44] Kenny P. W. Hydrogen-bond donors in drug design. J. Med. Chem. 2022, 65, 14261–14275. 10.1021/acs.jmedchem.2c01147. 36282210

[ref45] Kenny P. W.; Montanari C. A.; Prokopczyk I. M. Clog Palk: A method for predicting alkane/water partition coefficient. J. Comput.-Aided Mol. Des. 2013, 27, 389–402. 10.1007/s10822-013-9655-5. 23737238

[ref46] Beinat C.; Reekie T.; Banister S. D.; O’Brien-Brown J.; Xie T.; Olson T. T.; Xiao Y.; Harvey A.; O’Connor S.; Coles C.; Grishin A.; Kolesik P.; Tsanaktsidis J.; Kassiou M. Structure–activity relationship studies of SEN12333 analogues: Determination of the optimal requirements for binding affinities at α7 nAChRs through incorporation of known structural motifs. Eur. J. Med. Chem. 2015, 95, 277–301. 10.1016/j.ejmech.2015.03.025. 25827398

[ref47] Kabsch W. XDS. Acta Crystallogr., Sect. D: Biol. Crystallogr. 2010, 66, 125–132. 10.1107/S0907444909047337. 20124692 PMC2815665

[ref48] Navaza J. AMoRe: An automated package for molecular replacement. Acta Crystallogr., Sect. A: Found. Crystallogr. 1994, 50, 157–163. 10.1107/S0108767393007597.

[ref49] Xing Y.; Clements W. K.; Le Trong I.; Hinds T. R.; Stenkamp R.; Kimelman D.; Xu W. Crystal Structure of a beta-Catenin/APC Complex Reveals a Critical Role for APC Phosphorylation in APC Function. Mol. Cell. 2004, 15, 523–533. 10.1016/j.molcel.2004.08.001. 15327769

[ref50] Brünger A. T.; Adams P. D.; Clore G. M.; DeLano W. L.; Gros P.; Grosse-Kunstleve R. W.; Jiang J. S.; Kuszewski J.; Nilges M.; Pannu N. S.; Read R. J.; Rice L. M.; Simonson T.; Warren G. L. Crystallography & NMR system: A new software suite for macromolecular structure determination. Acta Crystallogr., Sect. D: Biol. Crystallogr. 1998, 54, 905–921. 10.1107/s0907444998003254. 9757107

[ref51] Adams P. D.; Afonine P. V.; Bunkóczi G.; Chen V. B.; Davis I. W.; Echols N.; Headd J. J.; Hung L. W.; Kapral G. J.; Grosse-Kunstleve R. W.; McCoy A. J.; Moriarty N. W.; Oeffner R.; Read R. J.; Richardson D. C.; Richardson J. S.; Terwilliger T. C.; Zwart P. H. PHENIX: a comprehensive Python-based system for macromolecular structure solution. Acta Crystallogr., Sect. D: Biol. Crystallogr. 2010, 66, 213–221. 10.1107/S0907444909052925. 20124702 PMC2815670

[ref52] Jönsson U.; Fägerstam L.; Ivarsson B.; Johnsson B.; Karlsson R.; Lundh K.; Löfås S.; Persson B.; Roos H.; Rönnberg I. Real-time biospecific interaction analysis using surface plasmon resonance and a sensor chip technology. Biotechniques 1991, 11, 620–627. 1804254

[ref53] Madhavi Sastry G.; Adzhigirey M.; Day T.; Annabhimoju R.; Sherman W. Protein and ligand preparation: parameters, protocols, and influence on virtual screening enrichments. J. Comput. Aided Mol. Des. 2013, 27, 221–234. 10.1007/s10822-013-9644-8. 23579614

[ref54] PyMOL version1.2r1; DeLanoScientificLLC: SanCarlos, CA.

[ref55] Roehm N. W.; Rodgers G. H.; Hatfield S. M.; et al. An improved colorimetric assay for cell proliferation and viability utilizing the tetrazolium salt XTT. J. Immunol. Methods 1991, 142, 257–265. 10.1016/0022-1759(91)90114-u. 1919029

[ref56] Van Meerloo J.; Kaspers G. J. L.; Cloos J. Cell sensitivity assay: The MTT assay. Methods Mol. Biol. 2011, 731, 237–245. 10.1007/978-1-61779-080-5_20. 21516412

[ref57] Peng Y.; Qin Y.; Zhang X.; Deng S.; Yuan Y.; Feng X.; Chen W.; Hu F.; Gao Y.; He J.; Cheng Y.; Wei Y.; Fan X.; Ashktorab H.; Smoot D.; Li S.; Meltzer S. J.; Zhuang S.; Tang N.; Jin Z. MiRNA-20b/SUFU/Wnt axis accelerates gastric cancer cell proliferation, migration and EMT. Heliyon 2021, 7, e06695 10.1016/j.heliyon.2021.e06695. 33912703 PMC8065298

[ref58] Di Magno L.; Manni S.; Di Pastena F.; Coni S.; Macone A.; Cairoli S.; Sambucci M.; Infante P.; Moretti M.; Petroni M.; Nicoletti C.; Capalbo C.; De Smaele E.; Di Marcotullio L.; Giannini G.; Battistini L.; Goffredo B. M.; Iorio E.; Agostinelli E.; Maroder M.; Canettieri G. Phenformin inhibits Hedgehog-dependent tumor growth through a complex i-independent redox/corepressor module. Cell Rep. 2020, 30, 1735–1752. 10.1016/j.celrep.2020.01.024. 32049007

[ref59] Berardozzi S.; Bernardi F.; Infante P.; Ingallina C.; Toscano S.; De Paolis E.; Alfonsi R.; Caimano M.; Botta B.; Mori M.; Di Marcotullio L.; Ghirga F. Synergistic inhibition of the Hedgehog pathway by newly designed Smo and Gli antagonists bearing the isoflavone scaffold. Eur. J. Med. Chem. 2018, 156, 554–562. 10.1016/j.ejmech.2018.07.017. 30025349

[ref60] Spiombi E.; Angrisani A.; Fonte S.; De Feudis G.; Fabretti F.; Cucchi D.; Izzo M.; Infante P.; Miele E.; Po A.; Di Magno L.; Magliozzi R.; Guardavaccaro D.; Maroder M.; Canettieri G.; Giannini G.; Ferretti E.; Gulino A.; Di Marcotullio L.; Moretti M.; De Smaele E. KCTD15 inhibits the Hedgehog pathway in Medulloblastoma cells by increasing protein levels of the oncosuppressor KCASH2. Oncogenesis 2019, 8, 64 10.1038/s41389-019-0175-6. 31685809 PMC6828672

