# Correction to “Novel *N*‑(Heterocyclylphenyl)benzensulfonamide Sharing an Unreported Binding Site with T‑Cell Factor 4 at the β‑Catenin Armadillo Repeats Domain as Anticancer Agent”

**DOI:** 10.1021/acsptsci.5c00175

**Published:** 2025-03-20

**Authors:** Marianna Nalli, Laura Di Magno, Yichao Wen, Xin Liu, Michele D’Ambrosio, Michela Puxeddu, Anastasia Parisi, Jessica Sebastiani, Andrea Sorato, Antonio Coluccia, Silvia Ripa, Fiorella Di Pastena, Davide Capelli, Roberta Montanari, Domiziana Masci, Andrea Urbani, Chiara Naro, Claudio Sette, Viviana Orlando, Sara D’Angelo, Stefano Biagioni, Chiara Bigogno, Giulio Dondio, Arianna Pastore, Mariano Stornaiuolo, Gianluca Canettieri, Te Liu, Romano Silvestri, Giuseppe La Regina

We made corrections due to the fact that crystallographic and biological studies were conducted with two different compounds, using compounds **6** and **9**, respectively.

Page 1087, Table of Contents
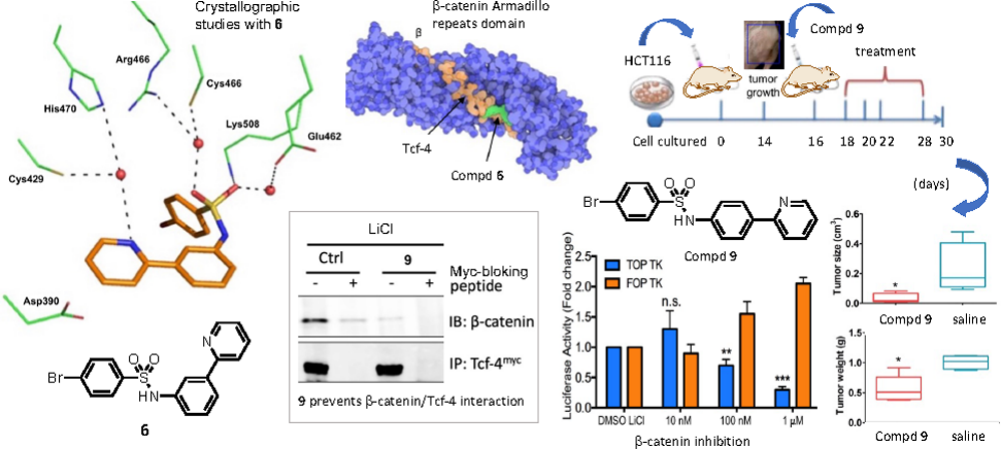


Page 1087, Abstract (from line 6 to the end). The crystallographic analysis of the β-catenin armadillo repeats domain revealed that compound **6** and Tcf-4 share a common binding site within the hotspot binding region close to Lys508. To our knowledge, compound **6** is the first small molecule ligand of this region to be reported. These results highlight the potential of this novel class of β-catenin inhibitors as anticancer agents.

Page 1088, column 2, from line 6 to the end. Compound **6** shared the binding site with Tcf-4 (PDB ID 2GL7) within the hotspot binding region close to Lys508 of the β-catenin armadillo repeats domain. Compound **9** abrogated the association between β-catenin and Tcf-4 in cells transfected with Myc-tagged Tcf-4. There is a substantial lack of structural data associated with the β-catenin inhibitors reported so far. For a number of inhibitors, the mechanism of action was inferred by the biological data;^21^ for others, just a putative binding area was proposed,^17^ and only for one derivative was reported an experimental binding mode.^22^ To our knowledge, **6** is the first small molecule ligand of this crucial region to be reported.

Page 1088, column 2, *Crystallographic studies. Binding of* **6** *in the β-catenin Armadillo repeats domain.* With the aim to elucidate the binding mode of **6** within the β-catenin Armadillo repeats domain...

Page 1089, column 1, line 8. In addition, the heterocycle ring of **6** forms...

Page 1089, column 1, line 13 to the end. A second water molecule bridges the side chains of His470 and Cys429 with the nitrogen heterocycle ring of **6**. Finally, a third water links the first sulfonamide oxygen to the side chain of Glu462. Docking proposed binding modes of derivative **6** and reference compound **3** are depicted in the Supporting Information, Figure S2.

Page 1089, Figure 2. Crystal structure of **6** in complex with the β-catenin Armadillo repeats domain. Panel A, superposition of the β-catenin Armadillo repeats domain in complex with Tcf-4 (orange; PDB ID 2GL7) and **6** (green; PDB ID 7ZRB). Panel B, Superposition of Tcf-4 and **6** within the “hotspot region 1” of the β-catenin Armadillo repeats domain. Figures were generated using the Protein Imager.^23^

Page 1090, Figure 3. Summary of the interactions between **6** and the β-catenin Armadillo repeats domain.

Page 1090, column 1, line 1. affinity of **6**...

Page 1090, column 1, line 11. Full kinetic analysis of the binding interaction between **6**...

Page 1090, column 1, line 15. ... rate constant of **6**...

Page 1090, Figure 4. SPR Kinetic analysis. Sensorgrams (left panel) and dose–response plot (right panel) of **6** binding to...

Page 1094, CONCLUSIONS columns 1, line 10 to the end. Compound **6** was chosen for the crystallographic studies. The crystal structure of compound **6** in complex the β-catenin Armadillo repeats domain was determined at 3.4 Å resolution from apoprotein crystals soaked with the ligand. Compound **6** occupied a solvent-exposed cavity resulting from the curvature of the alpha-solenoid superhelix involving the Armadillo repeats 7 to 10.

Page 1094, CONCLUSIONS columns 2, line 1 to line 11. Superposition of the β-catenin Armadillo repeats domain with **6** and Tcf-4 (PDB ID 2GL7) demonstrates that the two ligands share a common binding site within the hotspot binding region close to Lys508. Compound **6** represents the first small molecule to be described in such a crucial region of this domain. The binding affinity of **6** to the β-catenin Armadillo repeats domain was measured by Surface Plasmon Resonance biosensor technology. slow dissociation rate constant of **6** accounted for low nanomolar binding affinity constant and correlated with the hydrophobic and polar characteristics of this molecule.^44,45^ In coimmunoprecipitation study...

Page 1095, column 1, last line. Crystallographic studies revealed that **6**...

## Supplementary Material



## Notes

Corrections to Supporting Information (PDF)

