# Supplementary material for: Novel N-(Heterocyclylphenyl)benzensulfonamide Sharing an Unreported Binding Site with T-Cell Factor 4 at the β-Catenin Armadillo Repeats Domain as an Anticancer Agent
Source: ACS Pharmacol Transl Sci. 2023 Jul 3;6(7):1087–103. doi: 10.1021/acsptsci.3c00092 (PMC10353061; doi:10.1021/acsptsci.3c00092)
Supplement: Supplementary file 1 — pt3c00092_si_001.pdf [file pt3c00092_si_001.pdf]

Supporting Information

Novel *N*-(Heterocyclylphenyl)benzensulfonamide Sharing an Unreported Binding Site with T-cell Factor 4 at the β-Catenin Armadillo Repeats Domain as Anticancer Agent

Marianna Nalli,[†,#] Laura Di Magno,[¢,#] Yichao Wen,[§,#] Xin Liu,[¥,#] Michele D'Ambrosio,[†] Michela Puxeddu,[†] Anastasia Parisi,[†] Jessica Sebastiani,[†] Andrea Sorato,[†] Antonio Coluccia,[†] Silvia Ripa,[¢] Fiorella Di Pastena,[¢] Davide Capelli,[‡] Roberta Montanari,[‡] Domiziana Masci,[£] Andrea Urbani,[£] Chiara Naro,[£,ž] Claudio Sette,[£,ž] Viviana Orlando,[œ] Sara D'Angelo,[œ] Stefano Biagioni,[œ] Chiara Bigogno,[ƒ] Giulio Dondio,[ƒ] Arianna Pastore,[¶] Mariano Stornaiuolo,[¶] Gianluca Canettieri,[¢] Te Liu,[§,*] Romano Silvestri,[†,*], Giuseppe La Regina,[†]

[†] *Laboratory affiliated to Istituto Pasteur Italia – Fondazione Cenci Bolognetti, Department of Drug Chemistry and Technologies, Sapienza University of Rome, Piazzale Aldo Moro 5, I-00185 Rome, Italy*
[¢] *Laboratory affiliated to Istituto Pasteur Italia – Fondazione Cenci Bolognetti, Department of Molecular Medicine Sapienza University of Rome, Viale Regina Elena 291, I-00161 Rome, Italy*
[§] *Shanghai Geriatric Institute of Chinese Medicine, Shanghai University of Traditional Chinese Medicine, 365 South Xiangyang Road, 200031 Shanghai, China*
[¥] *Department of Dermatology, Yueyang Hospital of Integrated Traditional Chinese and Western Medicine, Shanghai University of Traditional Chinese Medicine, 200437 Shanghai, China*
[‡] *CNR – Institute of Crystallography, Via Salaria – km 29.300, 00015 Monterotondo, Rome, Italy*
[£] *Department of Basic Biotechnological Sciences, Intensivological and Perioperative Clinics, Catholic University of the Sacred Heart, Largo Francesco Vito 1, 00168 Rome, Italy*
œ Department of Biology and Biotechnologies "Charles Darwin", Piazzale Aldo Moro 5, I-00185 Roma, Italy
[ƒ] *Aphad SrL, Via della Resistenza 65, 20090 Buccinasco, Italy*
[¶] *Department of Pharmacy, University of Naples "Federico II", Via Domenico Montesano, 49, 80131 Naples, Italy*
[ž] *GSTeP-Organoids Research Core Facility, Fondazione Policlinico Universitario A. Gemelli, IRCCS, 00168 Rome, Italy*

Corresponding author email: romano.silvestri@uniroma1.it

Contents of Supporting Information

Figure S1. 2Fo-Fc electron density map of compound **9** countered at 0.8 σ
Figure S2. Docking proposed binding modes of derivative **9** and reference **3**.
Figure S3. Inhibition of MDA-MB-231 cells by compound **3**
Figure S4. Inhibition of MDA-MB-231 cells by compound **5**
Figure S5. Inhibition of MDA-MB-231 cells by compound **6**
Figure S6. Inhibition of MDA-MB-231 cells by compound **7**
Figure S7. Inhibition of MDA-MB-231 by compound **8**
Figure S8. Inhibition of MDA-MB-231 cells by compound **9**
Figure S9. Inhibition of MDA-MB-231 cells by compound **10**

Figure S10. Inhibition of MDA-MB-231 cells by compound iCRT3
Figure S11. Inhibition of MDA-MB-231 cells by compound ICG-001
Figure S12. Inhibition of HCC-1806-231 cells by compound **3**
Figure S13. Inhibition of HCC-1806-231 cells by compound **5**
Figure S14. Inhibition of HCC-1806-231 cells by compound **6**
Figure S15. Inhibition of HCC-1806-231 cells by compound **7**
Figure S16. Inhibition of HCC-1806-231 cells by compound **8**
Figure S17. Inhibition of HCC-1806-231 cells by compound **9**
Figure S18. Inhibition of HCC-1806-231 cells by compound **10**
Figure S19. Inhibition of HCC-1806-231 cells by compound ICG-001
Figure S20. Inhibition of HCC-1937 cells by compound **3**
Figure S21. Inhibition of HCC-1937 cells by compound **6**
Figure S22. HPLC chromatogram of compound **5**
Figure S23. HPLC chromatogram of compound **6**
Figure S24. HPLC chromatogram of compound **7**
Figure S25. HPLC chromatogram of compound **8**
Figure S26. HPLC chromatogram of compound **9**
Figure S27. HPLC chromatogram of compound **10**
Figure S28. HPLC chromatogram of reference compound **3**
Table S1. Statistics of crystallographic data and refinement for crystals of β-catenin Armadillo repeats domain in complex with **9**
Table S2. HPLC gradient profile A for compounds **3**, **5** and **8.**
Table S3. HPLC gradient profile B for compounds **6**, **7**, **9** and **10.**

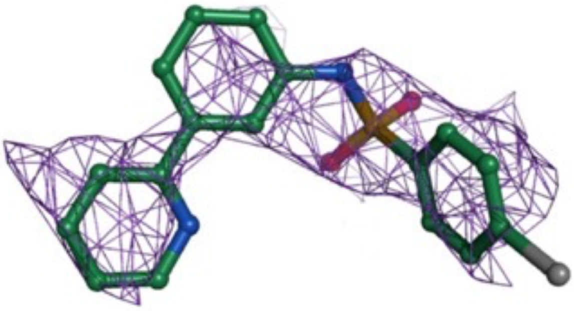

Figure S1. 2Fo-Fc electron density map of RS6452 countered at 0.8 σ

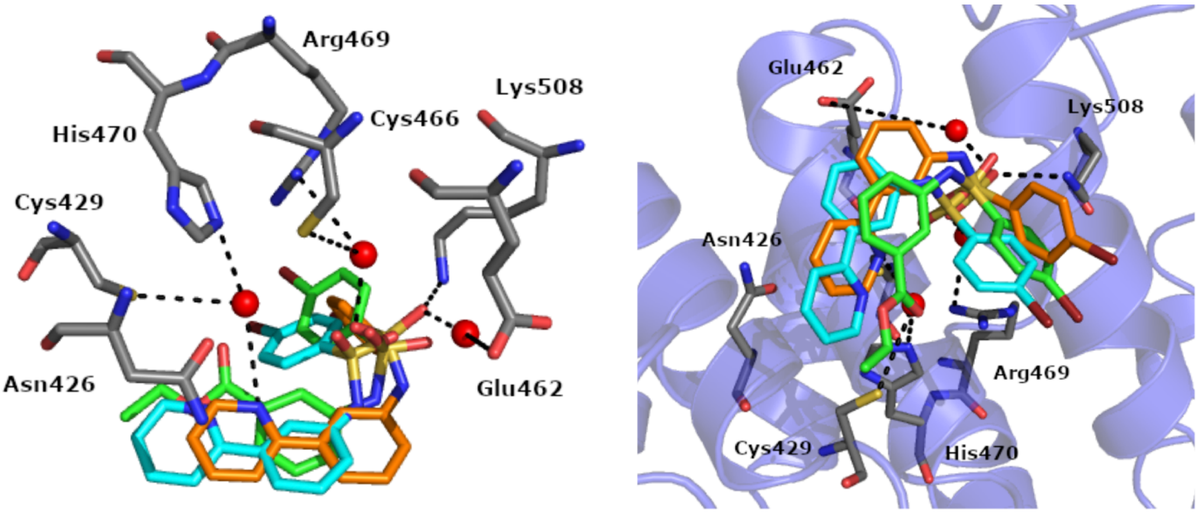

Figure S2. Docking proposed binding mode for derivatives **3** and **9**. The compounds are reported as stick green for **3**, cyan for docking pose of **9** and orange for the crystallographic pose of **9**. Residues involved in interactions are depicted as grey stick, water molecules are reported as red spheres. H-bonds contacts are showed as black dot lines. Protein is reported as light blue cartoon.

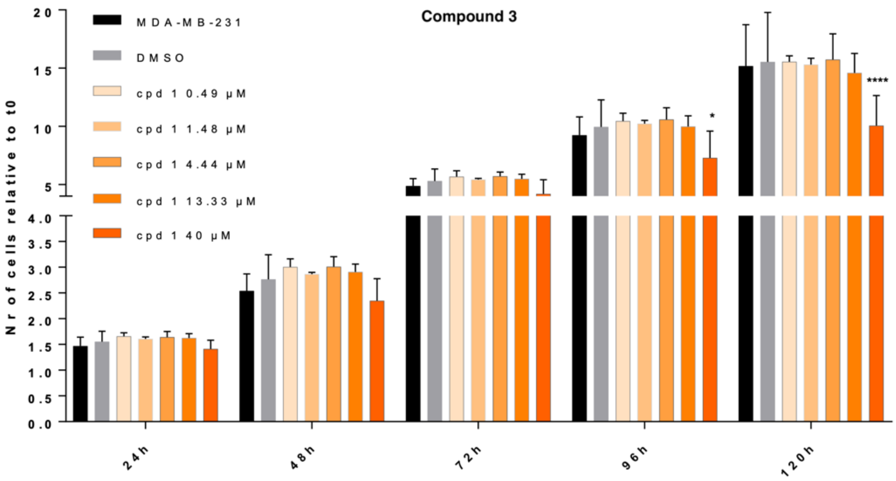

Figure S3. Inhibition of MDA-MB-231 cells by compound **3**. The number of cells at each time-point was quantified using the IncuCyte Cell-by-Cell analysis software and cell growth was evaluated as the ratio between counted cells and those counted at time 0 (mean ± SD, n=3, two-way Anova, $*p \leq 0.05$, $**p \leq 0.01$, $****p \leq 0.0001$,).

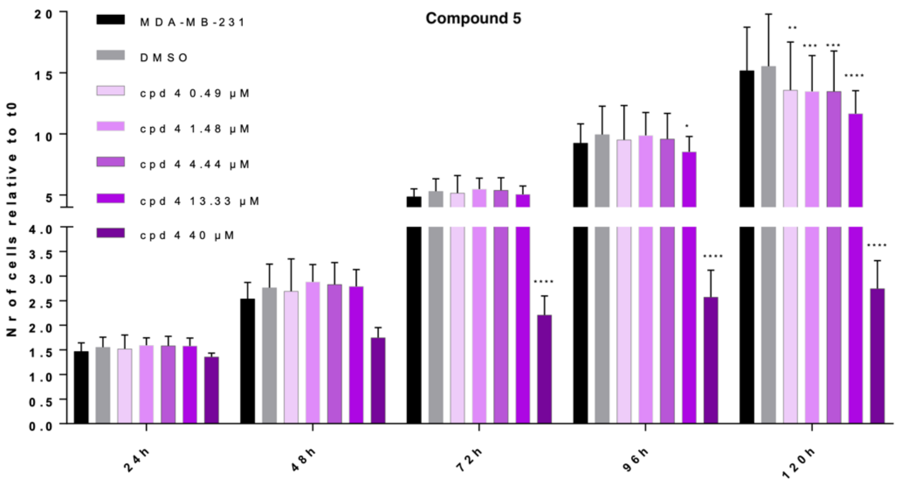

Figure S4. Inhibition of MDA-MB-231 cells by compound **5**. (See Figure S3).

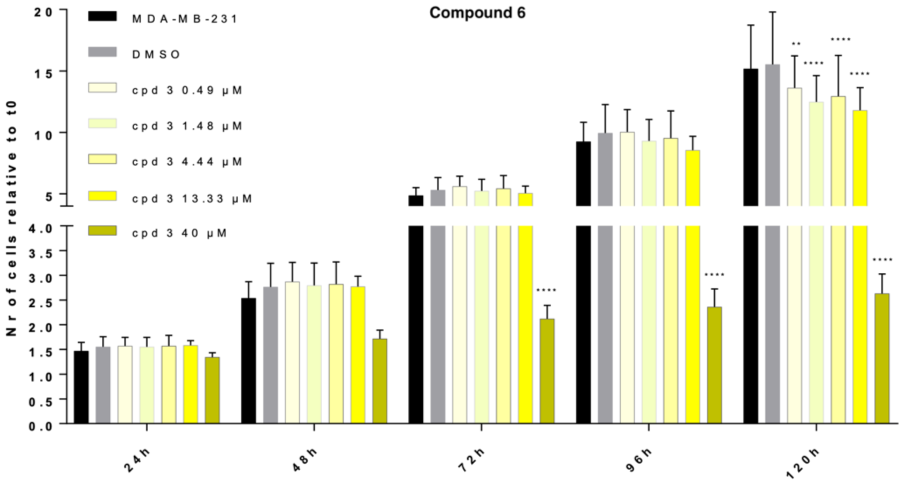

Figure S5. Inhibition of MDA-MB-231 cells by compound **6**. (See Figure S3).

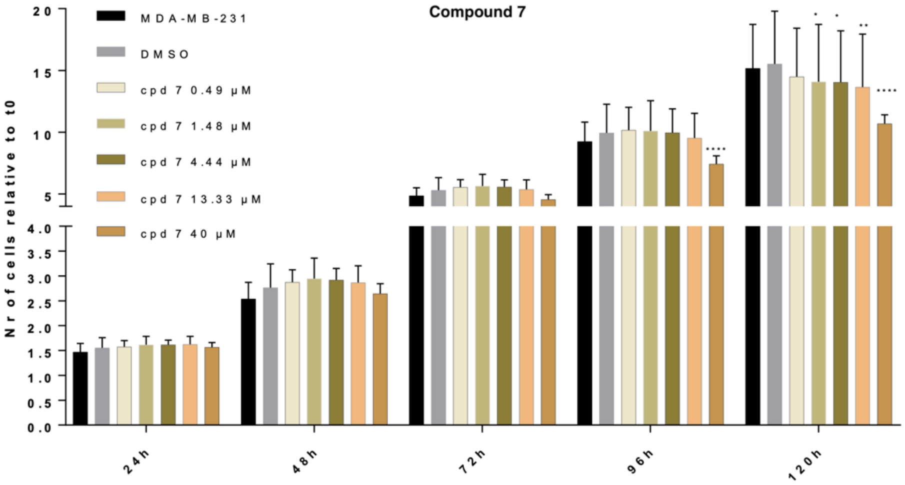

Figure S6. Inhibition of MDA-MB-231 cells by compound **7**. (See Figure S3).

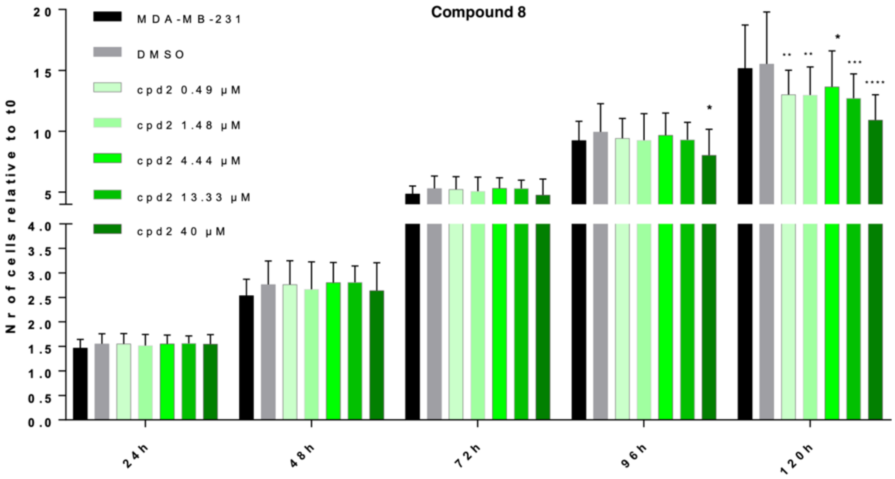

Figure S7. Inhibition of MDA-MB-231 cells by compound **8**. (See Figure S3).

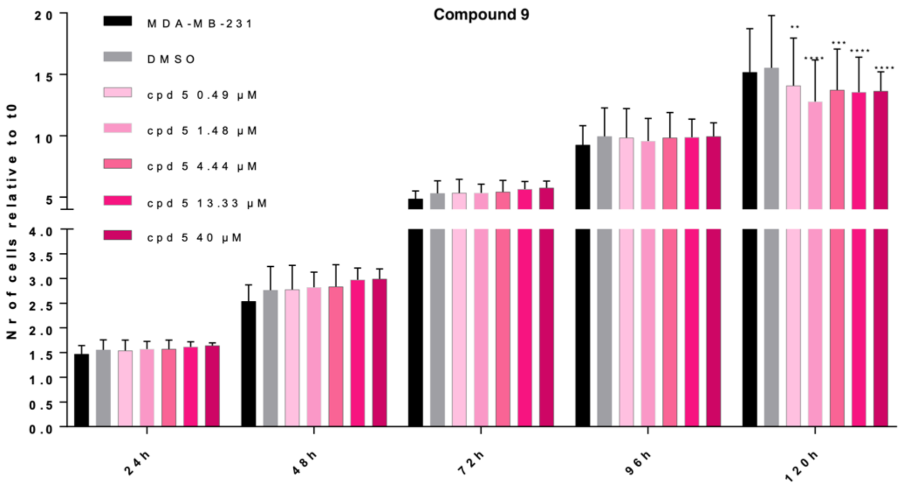

Figure S8. Inhibition of MDA-MB-231 cells by compound **9**. (See Figure S3).

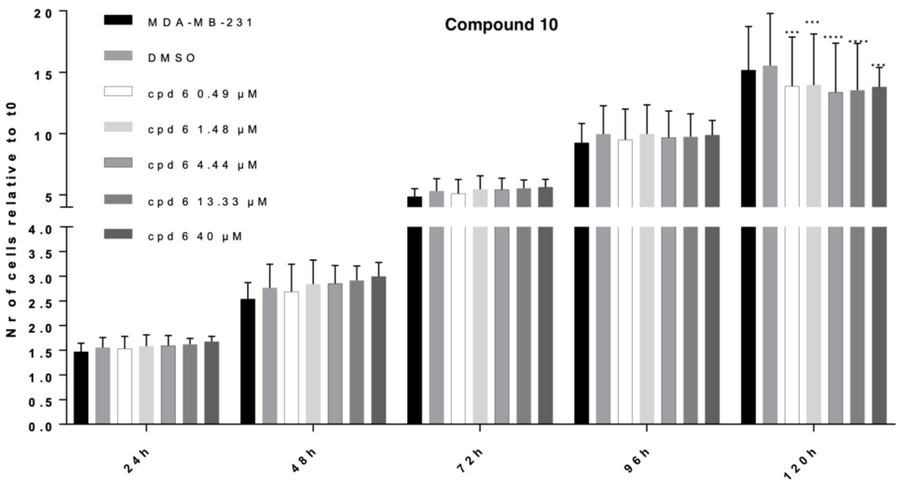

Figure S9. Inhibition of MDA-MB-231 cells by compound **10**. (See Figure S3).

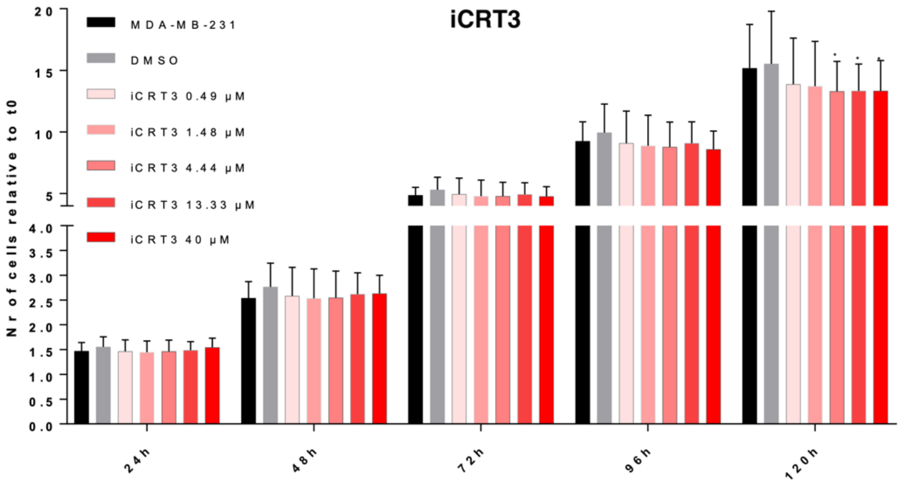

Figure S10. Inhibition of MDA-MB-231 cells by compound iCRT3. (See Figure S3).

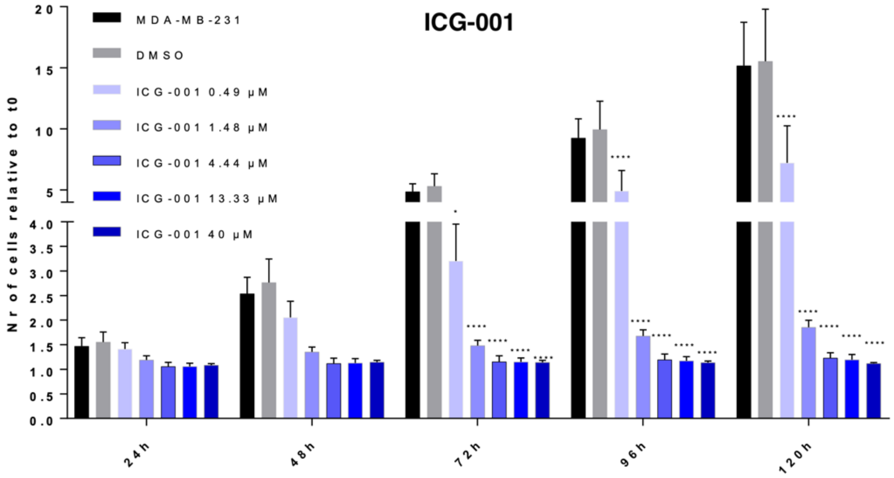

Figure S11. Inhibition of MDA-MB-231 cells by compound ICG-001. (See Figure S3).

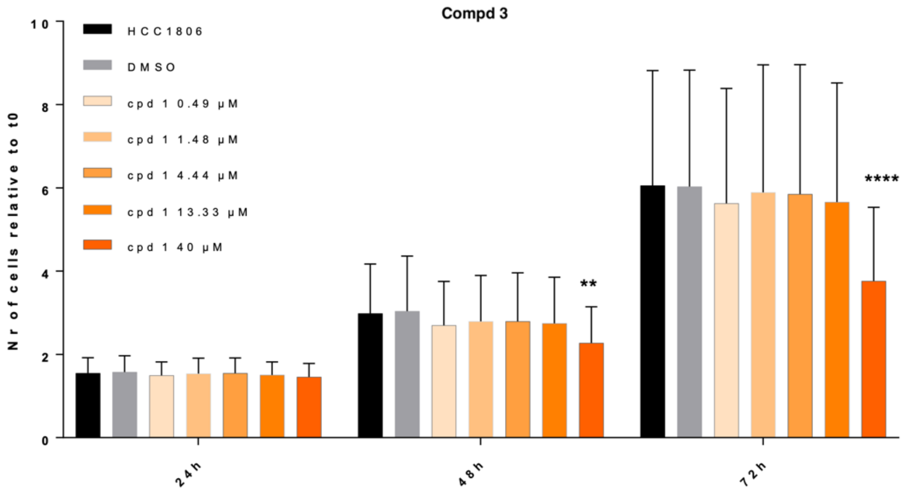

Figure S12. Inhibition of HCC-1806-231 cells by compound **3**. (See Figure S3).

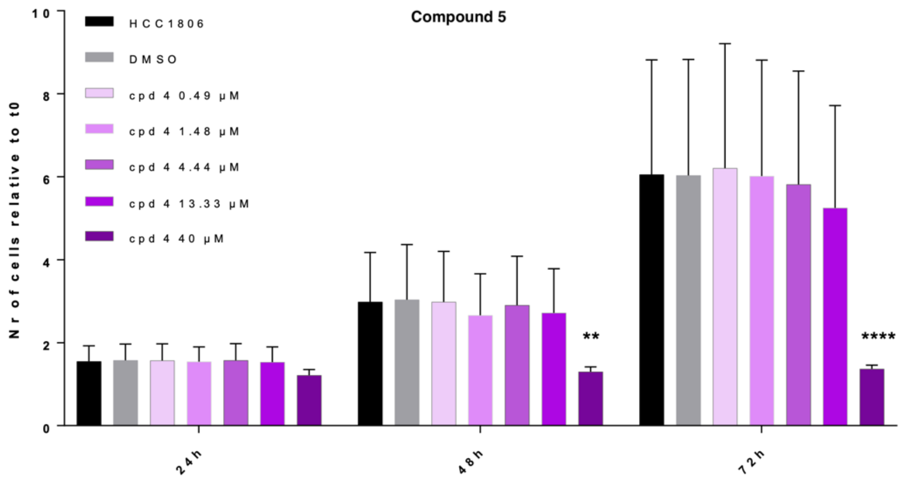

Figure S13. Inhibition of HCC-1806-231 cells by compound **5**. (See Figure S3).

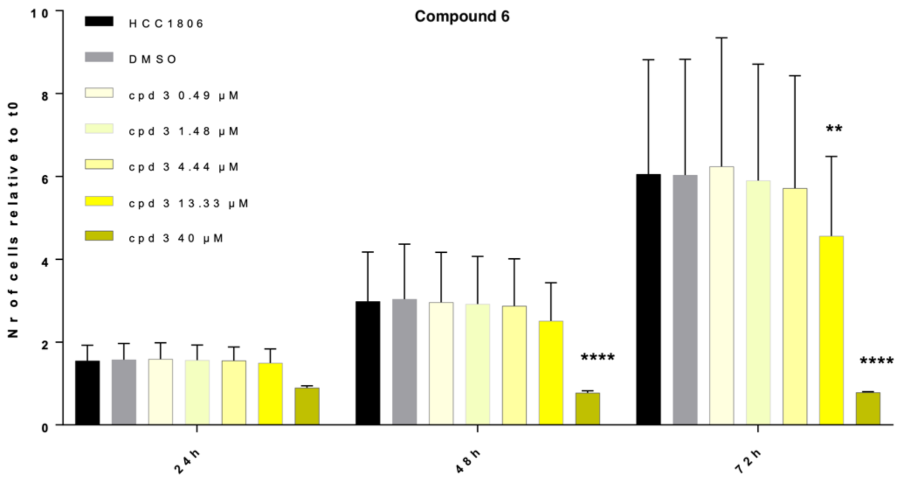

Figure S14. Inhibition of HCC-1806-231 cells by compound **6**. (See Figure S3).

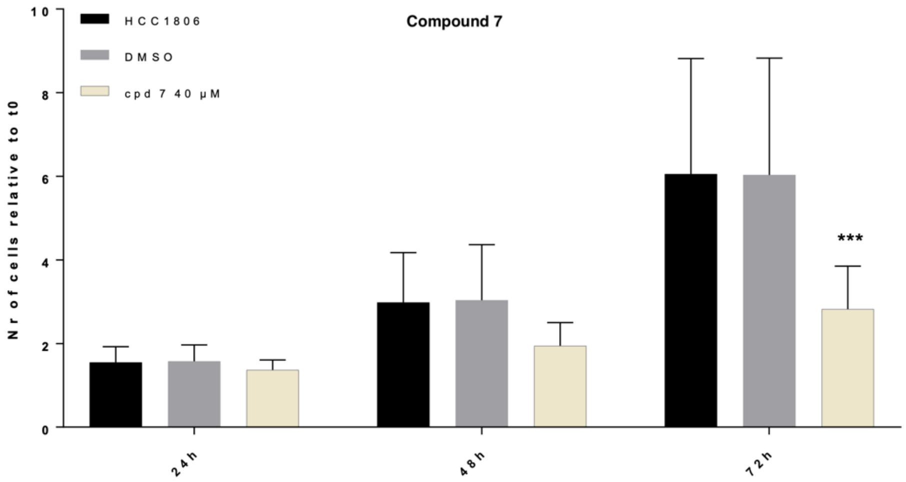

Figure S15. Inhibition of HCC-1806-231 cells by compound **7**. (See Figure S3).

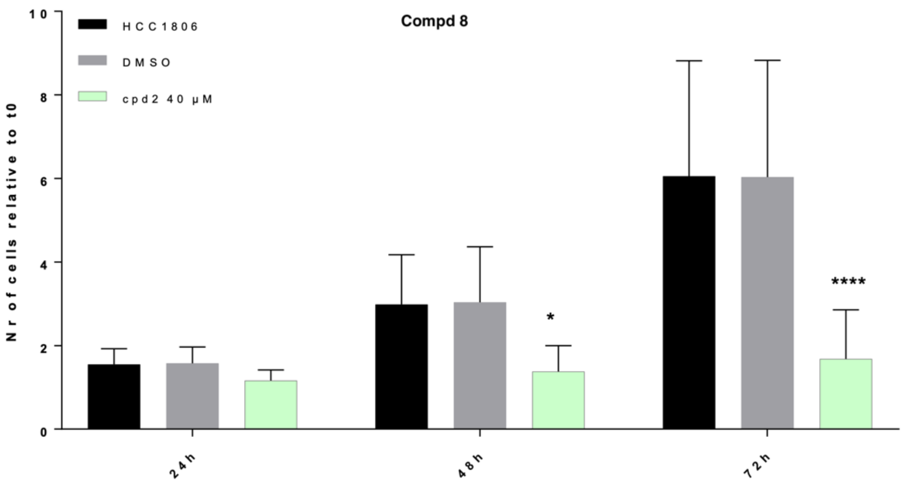

Figure S16. Inhibition of HCC-1806-231 cells by compound **8**. (See Figure S3).

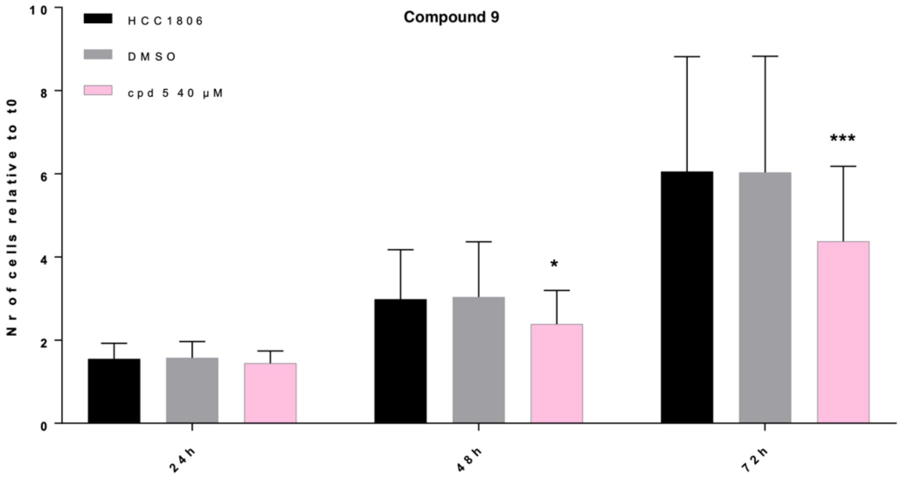

Figure S17. Inhibition of HCC-1806-231 cells by compound **9**. (See Figure S3).

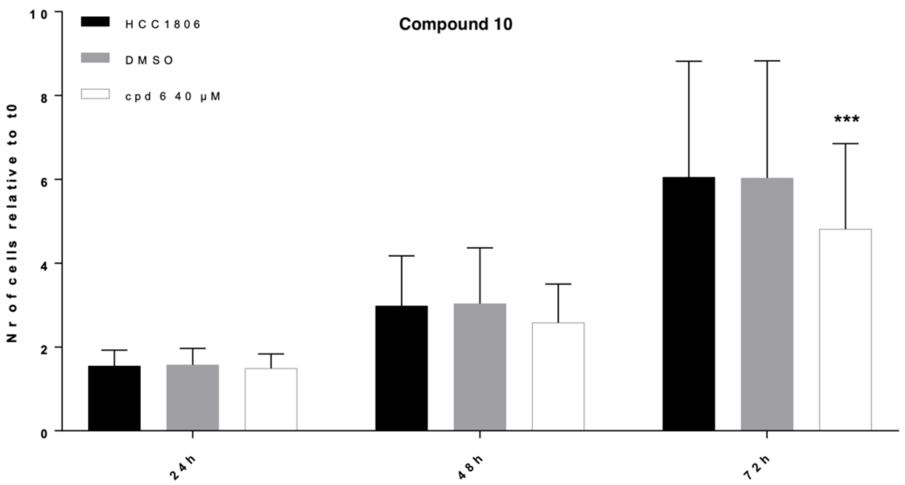

Figure S18. Inhibition of HCC-1806-231 cells by compound **10**. (See Figure S3).

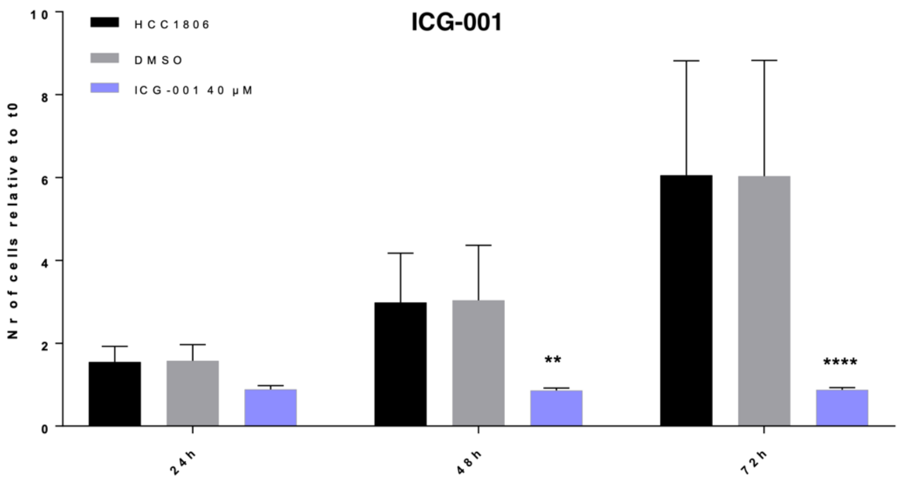

Figure S19. Inhibition of HCC-1806-231 cells by compound ICG-001. (See Figure S3).

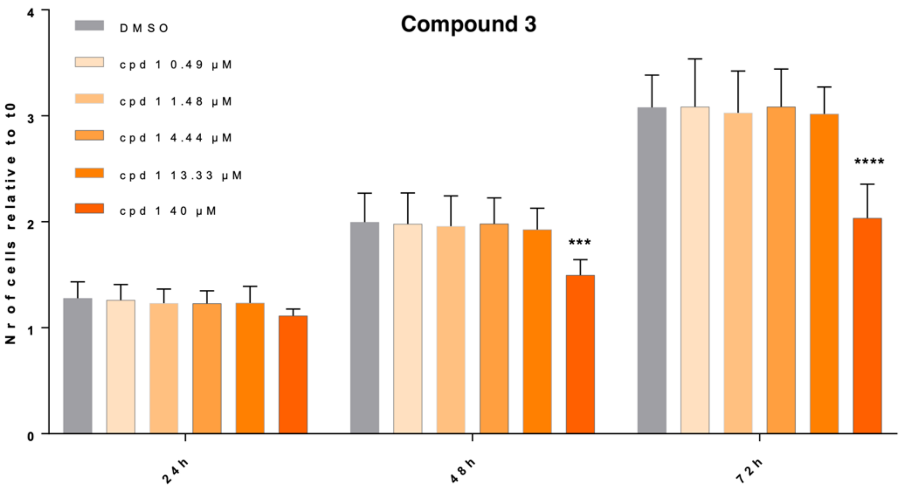

Figure S20. Inhibition of HCC-1937 cells by compound **3**. (See Figure S3).

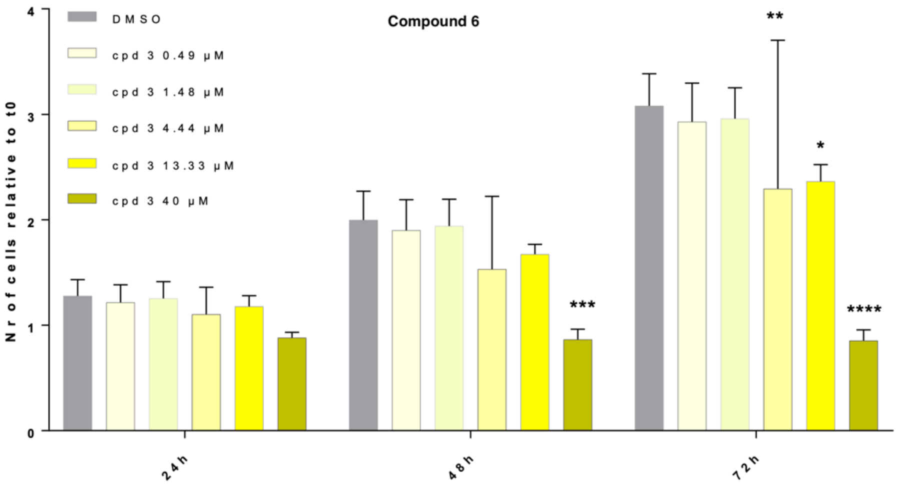

Figure S21. Inhibition of HCC-1937 cells by compound **6**. (See Figure S3).

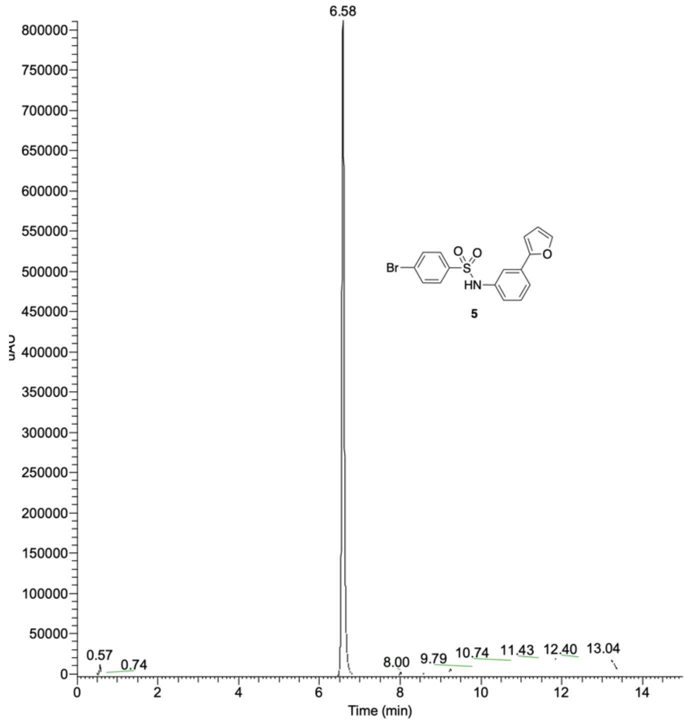

Figure S22. HPLC chromatogram of compound **5**.

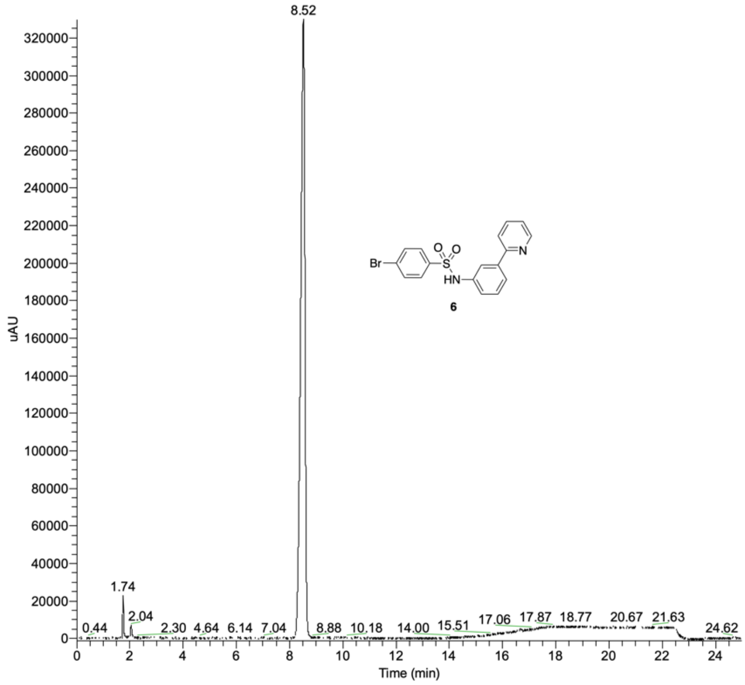

Figure S23. HPLC chromatogram of compound **6**.

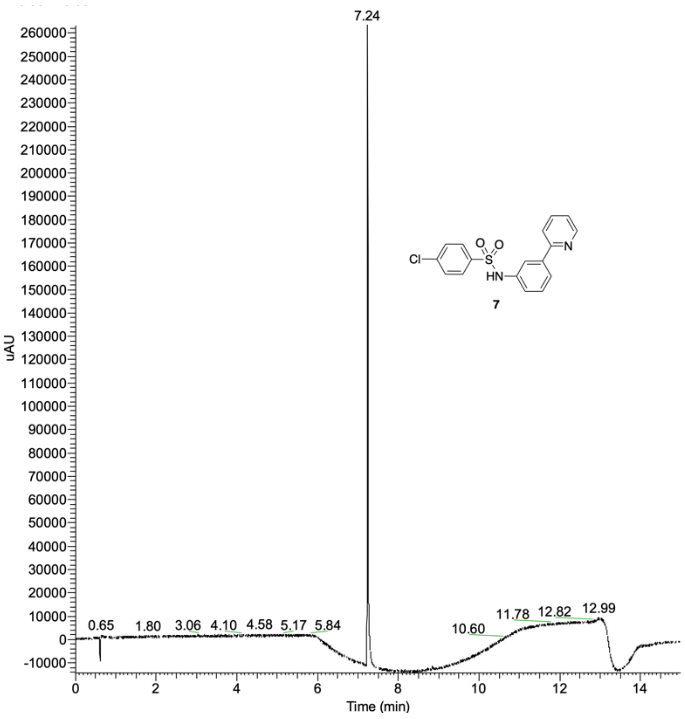

Figure S24. HPLC chromatogram of compound **7**.

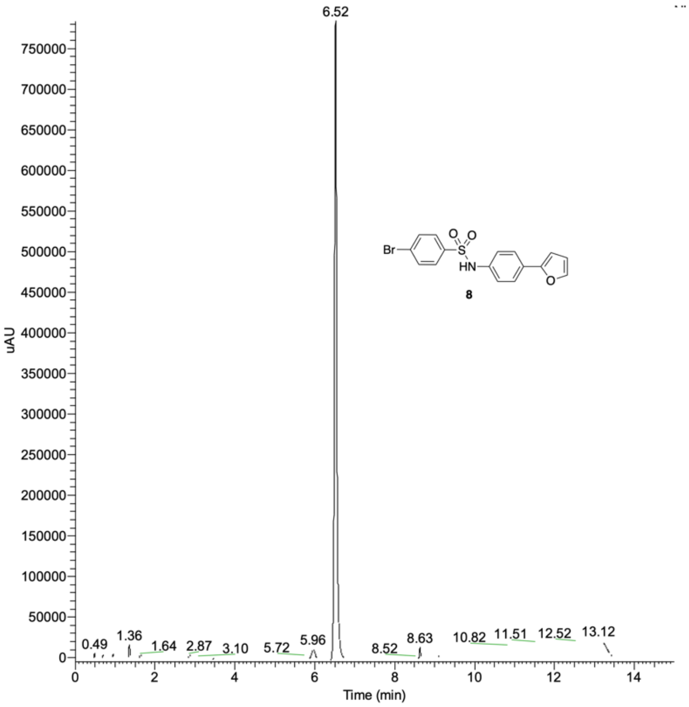

Figure S25. HPLC chromatogram of compound **8**.

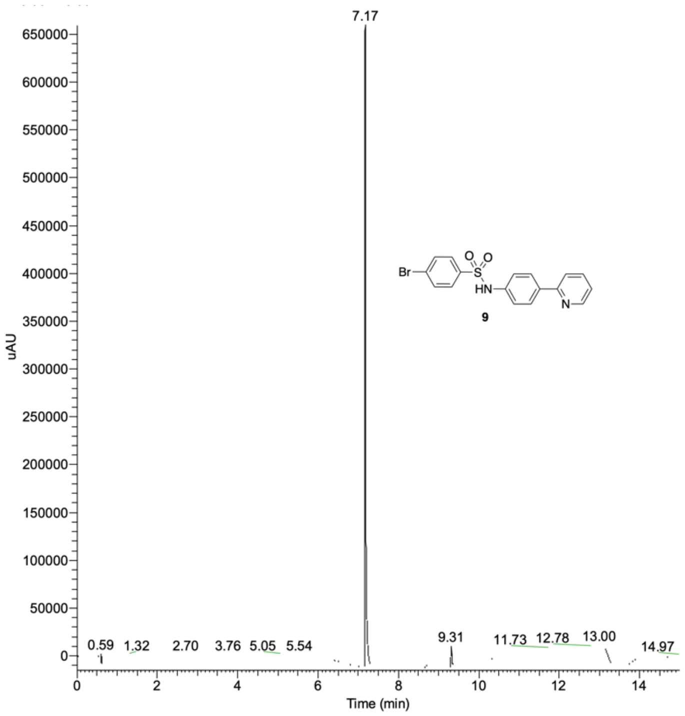

Figure S26. HPLC chromatogram of compound **9**.

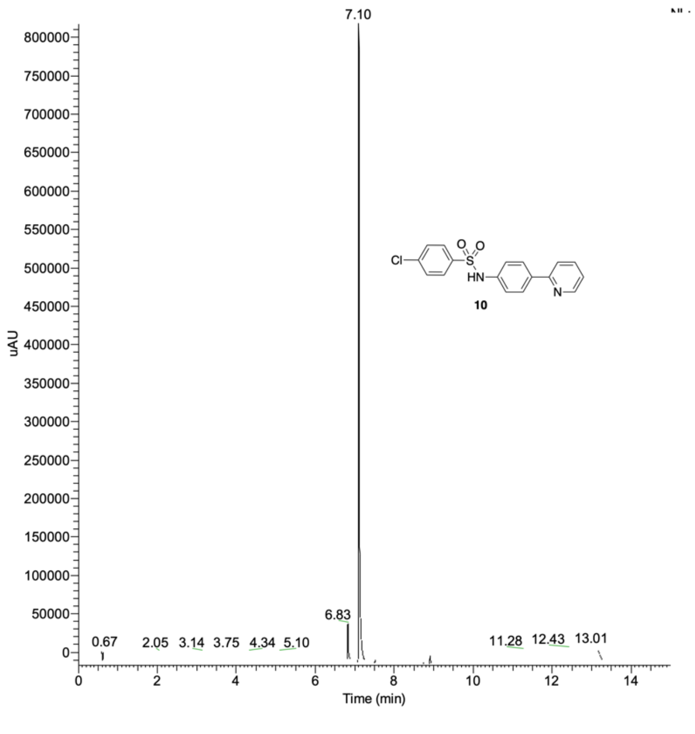

Figure S27. HPLC chromatogram of compound **10**.

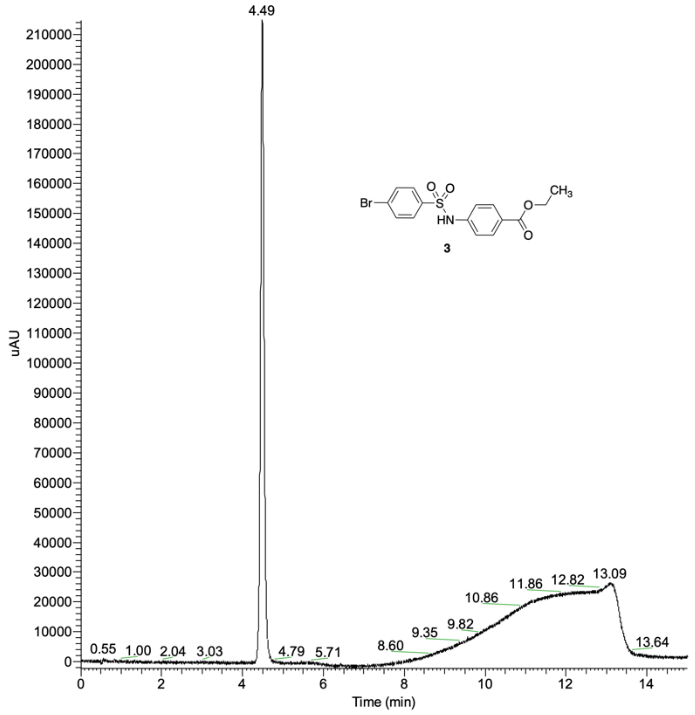

Figure S28. HPLC chromatogram of compound **3**.

Table 1S. Statistics of crystallographic data and refinement for crystals of β-catenin Armadillo repeats domain in complex with **9**.

	**Armadillo/9**
**Data collection**	
space group	*P2(1)*
cell dimension a, b, c [Å]	49.88, 160.24, 79.97
wavelenght [Å]	1.0
resolution range [Å]	78.84 - 3.43
last shell [Å]	3.66 - 3.43
R_{merge} [%]	21.9 (188)[a]
unique reflections	12910 (646)[a]
mean $(I)/\sigma(I)$	7.3 (1.0)[a]
Completeness (ellipsoidal)	91.2 (50.0)[a]
N. of molecules in asymmetric unit	2
**Refinement**	
resolution range [Å]	50.00 - 3.43
R_{work} [%]	23.25
R_{free} [%]	28.33
Bond lenghts r.m.s.d. [Å]	0.003
Bond angles r.m.s.d. [deg]	0.653

[a]The values in parenthesis refer to the outer shell.

Table S2. HPLC gradient profile A for compounds **3**, **5** and **8.**[a]

min	% A	% B
0	60	40
5	60	40
10	0	100
12	0	100
13	60	40
15	60	40

Table S3. HPLC gradient profile B for compounds **6**, **7**, **9** and **10.**[a]

min	% A	% B
0	90	10
5	90	10
10	0	100
12	0	100
13	90	10
15	90	10

[a] Eluents: A) H_2O/ACN, 95/5 + 0,1 % trifluoroacetic acid; B) ACN + 0,1 % trifluoroacetic acid.
