# Supplementary material for: Correction to “Novel N‑(Heterocyclylphenyl)benzensulfonamide Sharing an Unreported Binding Site with T‑Cell Factor 4 at the β‑Catenin Armadillo Repeats Domain as Anticancer Agent”
Source: ACS Pharmacol Transl Sci. 2025 Mar 20;8(4):1185–6. doi: 10.1021/acsptsci.5c00175 (PMC11997877; doi:10.1021/acsptsci.5c00175)
Supplement: Supplementary file 1 [file pt5c00175_si_001.pdf]

Corrections to Supporting Information

Title. “Novel N-(Heterocyclylphenyl)benzensulfonamide Sharing an Unreported Binding Site with T-cell Factor 4 at the β-Catenin Armadillo Repeats Domain as Anticancer Agent”

Author names. Marianna Nalli, Laura Di Magno, Yichao Wen, Xin Liu, Michele D’Ambrosio, Michela Puxeddu, Anastasia Parisi, Jessica Sebastiani, Andrea Sorato, Antonio Coluccia, Silvia Ripa, Fiorella Di Pastena, Davide Capelli, Roberta Montanari, Domiziana Masci, Andrea Urbani, Chiara Naro, Claudio Sette, Viviana Orlando, Sara D’Angelo, Stefano Biagioni, Chiara Bigogno, Giulio Dondio, Arianna Pastore, Mariano Stornaiuolo, Gianluca Canettieri, Te Liu, Romano Silvestri, Giuseppe La Regina

Journal citation. ACS Pharmacol. Transl. Sci. 2023, 6, 1087-1103

DOI. https://doi.org/10.1021/acsptsci.3c00092

**Description of the corrections to Supporting Information**

**Figure S1.** 2Fo-Fc electron density map of compound **6** countered at 0.8 σ
**Figure S2**. Docking proposed binding modes of derivative **6** and reference **3**.

**Figure S1.** 2Fo-Fc electron density map of **6** countered at 0.8 σ

**Figure S2.** Docking proposed binding mode for derivatives **3** and **6**. The compounds are reported as stick green for **3**, cyan for docking pose of **6** and orange for the crystallographic pose of **6**.
